# Urbanization shapes the landscape of antibiotic resistance genes and water quality microbial markers across an urban watershed

**DOI:** 10.3389/fmicb.2026.1974267

**Published:** 2026-10-09

**Authors:** Mary Bustamante, Somtopchukwu Opara, Ali Hammoud, Jared Schneider, Kara Bowles, Keshav Pokhrel, Jacob A. Napieralski, Sonia M. Tiquia-Arashiro

**Affiliations:** 1 Department of Natural Sciences, University of Michigan-Dearborn, Dearborn, MI, United States; 2 Department of Biology, Spring Arbor University, Spring Arbor, MI, United States; 3 Department of Biology, Northern Michigan University, Marquette, MI, United States; 4 Department of Mathematics and Statistics, University of Michigan-Dearborn, Dearborn, MI, United States

**Keywords:** antibiotic resistance genes, environmental antimicrobial resistance, freshwater microbiology, geospatial analysis, high-throughput quantitative PCR, One Health, Rouge River, watershed surveillance

## Abstract

Urbanization alters watershed hydrology, land use, and water quality, potentially influencing the distribution of antibiotic resistance genes (ARGs) and microbial contaminants in urban rivers. However, watershed-scale relationships among urbanization, microbial water quality markers, and environmental antimicrobial resistance remain incompletely understood. This study investigated spatial patterns and differences among sampling campaigns in ARGs and microbial water quality markers across the Rouge River watershed in southeastern Michigan, USA, by integrating high-throughput quantitative PCR (qPCR) arrays with watershed-scale geospatial characteristics, physicochemical measurements, and multivariate analyses. Water samples were collected from six sites spanning an urbanization gradient during three sampling campaigns (2023, 2024, and 2026). Detection profiles from arrays targeting 83 ARGs and 50 microbial water quality markers were evaluated using non-metric multidimensional scaling (NMDS), redundancy analysis (RDA), and exploratory Pearson correlations. Microbial water quality marker composition exhibited substantial variation among sites and sampling campaigns but was not significantly associated with the measured watershed characteristics or physicochemical variables. In contrast, ARG composition showed exploratory associations with several measured watershed and physicochemical gradients in the RDA, although the overall relationship was not statistically significant after accounting for repeated observations within sites. β-Lactam resistance genes comprised the largest proportion of detected ARGs, whereas tetracycline resistance genes exhibited the highest normalized detection frequency. Notably, the midstream Inkster site exhibited the greatest ARG richness in both 2024 and 2026 despite having an intermediate proportion of developed land. This repeated pattern occurred under contrasting antecedent precipitation conditions, suggesting that developed land and recent rainfall alone are unlikely to account for the observed ARG hotspot. These findings demonstrate that microbial water quality markers and ARGs exhibit distinct spatial patterns within the same urban watershed and provide new insight into the spatial complexity of environmental antimicrobial resistance in freshwater systems. Together, they underscore the value of integrated molecular, environmental, and geospatial approaches for watershed-scale AMR surveillance within a One Health framework.

## 1 Introduction

Urban watersheds are among the most heavily modified ecosystems worldwide, where increasing urbanization alters hydrology, land use, and water quality through stormwater runoff, wastewater discharges, industrial activities, and expanding human populations (Oest et al., 2018; Patel et al., 2019). These anthropogenic pressures transform urban waterways into reservoirs and transport pathways for microbial contaminants and antibiotic resistance genes (ARGs), increasing concerns about the environmental dissemination of antimicrobial resistance (Morina and Franklin, 2023; Nassereddine et al., 2024). As antimicrobial resistance (AMR) continues to emerge as a major global public health threat, understanding how watershed characteristics and urban-associated environmental pressures influence the distribution of ARGs and microbial contaminants has become a priority within the One Health framework, which recognizes the interconnectedness of human, animal, and environmental health (World Health Organization, 2026).

Aquatic environments are increasingly recognized as reservoirs for the persistence and dissemination of ARGs. Wastewater discharges, combined sewer overflows, stormwater runoff, industrial effluents, and other non-point pollution sources introduce antibiotic-resistant bacteria, mobile genetic elements, residual antibiotics, and other selective agents into receiving waters, facilitating the maintenance and spread of antimicrobial resistance (Marti et al., 2014; Morina and Franklin, 2023; Nassereddine et al., 2024). Urban waters also receive microbial contaminants originating from human wastewater, domestic animals, wildlife, agricultural runoff, and aging sewer infrastructure, resulting in complex mixtures of contamination sources (Harwood et al., 2014; Lobos et al., 2024). Although conventional monitoring relies primarily on fecal indicator bacteria such as Escherichia coli and enterococci, these indicators provide limited information regarding contamination sources and the broader microbial community (Harwood et al., 2014). Recent advances in high-throughput molecular technologies, including quantitative PCR (qPCR) arrays, enable the simultaneous detection of microbial source-tracking markers and ARGs, providing a more comprehensive assessment of microbial contamination and environmental health risks than conventional indicator-based monitoring (Nassereddine et al., 2024; Qyteti et al., 2025; Tiquia-Arashiro et al., 2025a).

The Rouge River watershed in southeastern Michigan provides an ideal model system for investigating relationships among urbanization, microbial water quality, and environmental antimicrobial resistance. Draining one of the most densely urbanized regions of the Great Lakes basin, the watershed encompasses a pronounced gradient from relatively less developed upstream areas to highly urbanized downstream reaches characterized by residential, commercial, industrial, and transportation land uses (Napieralski et al., 2015). Previous investigations have documented diverse microbial populations and spatial variation in microbial community characteristics, metabolic diversity, microbial activities, and ecosystem function throughout the Rouge River watershed, including bacterial adaptations to environmental stressors and contaminants, with these studies collectively demonstrating the influence of urbanization and anthropogenic conditions on riverine microbial systems (Tiquia et al., 2007, 2008; Tiquia, 2010a,b, 2011). Related studies of urban river and lake watersheds have also documented spatial variation in microbial metabolic function and carbon utilization, further demonstrating the sensitivity of aquatic microbial communities to watershed conditions (Patel et al., 2025). Despite substantial restoration efforts over the past several decades, the watershed continues to experience water quality impairment associated with urban runoff, wastewater inputs, and other anthropogenic stressors (Hartig and Graham, 2022). These characteristics make the Rouge River a useful system for investigating watershed-scale relationships among urbanization, microbial contamination, and environmental antimicrobial resistance.

Despite growing interest in environmental antimicrobial resistance, important knowledge gaps remain regarding the watershed-scale processes associated with the distribution of ARGs and microbial contamination in urban rivers. Although advances in high-throughput molecular technologies have greatly improved the detection of ARGs and microbial source-tracking markers, these approaches are often applied independently or within limited spatial and temporal sampling frameworks (Nassereddine et al., 2024). Consequently, relationships among watershed characteristics, physicochemical conditions, microbial contamination, and ARG occurrence remain incompletely understood at the watershed scale. Integrative approaches combining molecular profiling with environmental and spatial information have increasingly been recognized as important for tracking antibiotic resistance in aquatic ecosystems (Book et al., 2025). However, studies integrating high-throughput molecular profiling with geospatial analyses and environmental measurements across multiple sampling locations and years are still needed to determine whether ARGs and microbial water quality markers respond similarly to watershed-scale environmental gradients.

In this study, we integrated high-throughput molecular profiling using the Microbial DNA Antibiotic Resistance Genes Array and the Microbial DNA Water Quality Array with watershed-scale geospatial analyses and physicochemical measurements to investigate the occurrence, distribution, and environmental associations of ARGs and microbial water quality markers across the Rouge River watershed. Water samples collected during three sampling campaigns (2023, 2024, and 2026) from six sites representing an urbanization gradient were analyzed to (i) characterize the occurrence, richness, and distribution of ARGs and microbial water quality markers; (ii) evaluate spatial and temporal patterns along the urbanization gradient; (iii) examine relationships between watershed characteristics, physicochemical conditions, and molecular profiles; and (iv) assess the utility of integrating molecular, environmental, and geospatial data for watershed-scale surveillance. By directly comparing ARG and microbial water quality marker profiles from the same samples within a common environmental and multivariate framework, this study provides a multi-year assessment of microbial water quality and environmental antimicrobial resistance across an urban watershed and evaluates the complementary value of these molecular indicators for integrated watershed surveillance within a One Health framework (Tiquia-Arashiro et al., 2025a).

## 2 Materials and methods

### 2.1 Study area, sampling sites, and watershed characterization

The Rouge River watershed is located in southeastern Michigan, USA, and drains approximately 1,210 km^2^. It is among the most urbanized watersheds in the great lakes basin and spans a land-use gradient from relatively less-developed headwaters to densely developed downstream reaches characterized by residential, commercial, industrial, and transportation land uses. Six sampling sites were selected to provide spatial coverage from upstream to downstream, represent different watershed reaches and land-use settings along the urbanization gradient, maintain continuity with established rouge river sampling locations, and provide safe and practical access for repeated water collection (Figure 1). The sampling locations were Canton (Canton Lower Rouge Trail Way; 42.28139° N, 83.46586° W), Venoy (Venoy-Dorsey Park; 42.28854° N, 83.35928° W), Inkster (Inkster Park; 42.29872° N, 83.30706° W), Ford Field (Ford Field Park; 42.31189° N, 83.24300° W), Dearborn (Rouge River Gateway Trail–UM-Dearborn; 42.31068° N, 83.23165° W), and Rouge Park (42.36219° N, 83.25609° W). Site-specific landscape and hydrologic characteristics are summarized in Table 1.

**FIGURE 1:**
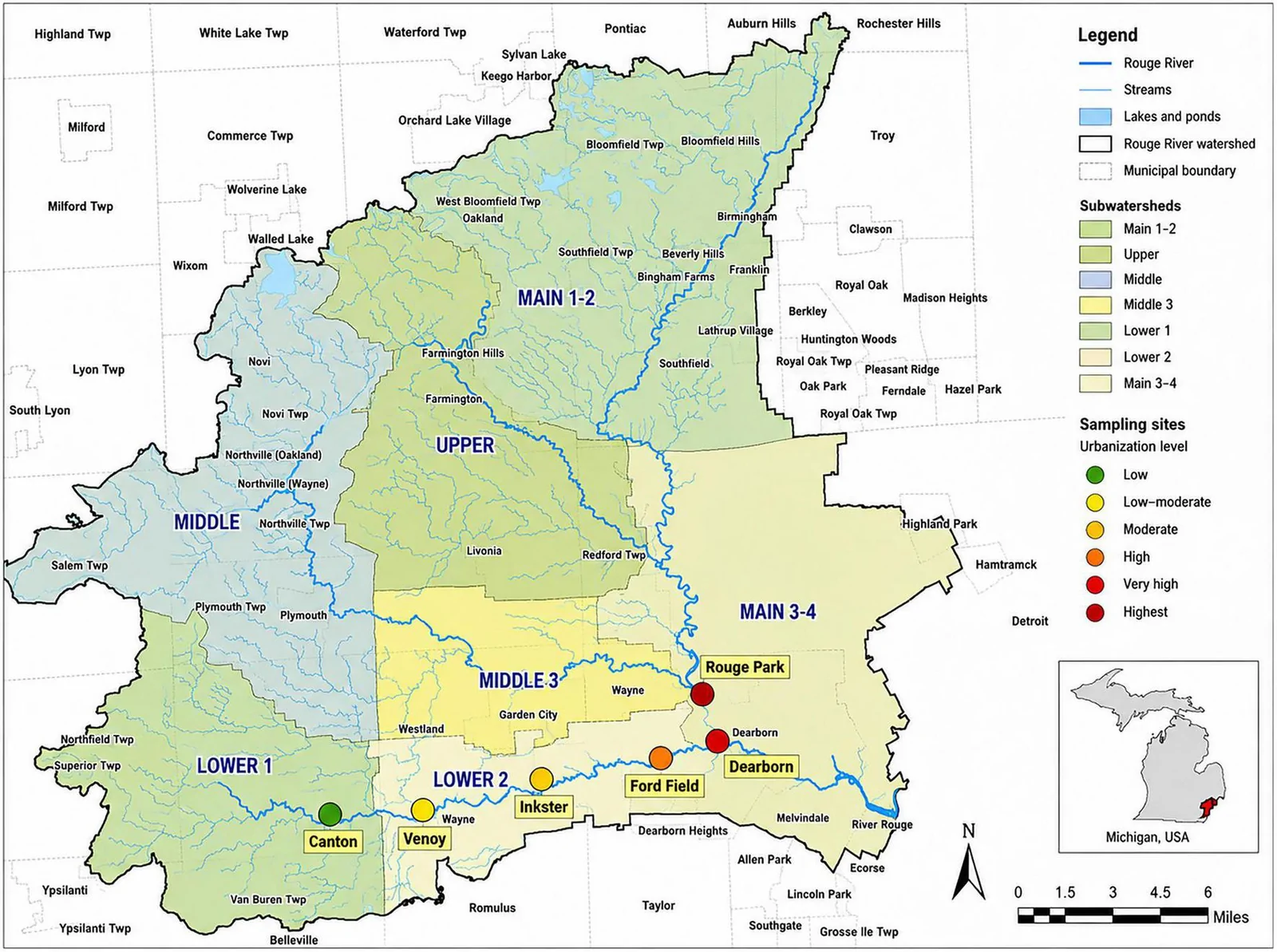
Map of the Rouge River watershed in southeastern Michigan, USA, showing six sampling locations distributed from upstream to downstream along the Rouge River: Canton, Venoy, Inkster, Ford Field, Dearborn, and Rouge Park. The legend reports developed land within the contributing watershed of each site as 46.3%, 61.0%, 63.0%, 67.5%, 67.4%, and 92.7%, respectively. The map also shows seven color-coded sub-watersheds, the Rouge River and stream network, lakes and ponds, municipal boundaries, a north arrow, a scale bar in kilometers, and a locator map of Michigan. Study area and sampling locations in the Rouge River watershed, southeastern Michigan, USA. Six sampling locations (Canton, Venoy, Inkster, Ford Field, Dearborn, and Rouge Park) are shown along the Rouge River from upstream to downstream. Sampling-site symbols are color coded, and the percentage of developed land within the contributing watershed of each site is provided in the legend as a quantitative indicator of watershed urbanization. The map also shows the Rouge River watershed boundary, major sub-watersheds, river and stream networks, lakes and ponds, municipal boundaries, and a locator map of Michigan.

**TABLE 1:** GIS-derived watershed characteristics of the six sampling sites along the Rouge River urbanization gradient.

Site	River Reach	Elevation (m)	Mean slope (%)	Total developed land (%)	Deciduous forest (%)	Cropland (%)	Stream density (km km^–^^2^)	Stream order	Soil group
Canton	Upstream	226.4	1.1	46.30	16.51	25.21	1.040	3	C/D
Venoy	Upper-midstream	219.2	0.9	60.98	14.00	13.46	1.090	4	C/D
Inkster	Midstream	217.0	0.9	63.04	13.37	12.40	1.050	4	C/D
Ford Field	Lower-midstream	214.1	0.8	67.47	12.07	10.92	0.993	4	C
Dearborn	Downstream	214.0	0.8	67.40	12.10	10.89	0.995	4	C
Rouge Park	Downstream	234.4	1.9	92.70	3.44	0.01	0.787	5	C

Total developed land (%) was calculated as the sum of developed open space and low-, medium-, and high-intensity developed land classes. GIS-derived landscape and hydrologic characteristics were obtained using ArcGIS Pro and the WikiWatershed platform. Stream order was classified using the Strahler system, in which increasing order indicates progressively larger channels formed by the convergence of upstream tributaries. Hydrologic soil groups follow the USDA Natural Resources Conservation Service classification. Group C soils have low infiltration rates following saturation, whereas Group D soils have very low infiltration rates and high runoff potential. Dual-group C/D soils behave as Group C under adequately drained conditions and as Group D under natural or poorly drained conditions.

Geospatial analyses were performed using ArcGIS Pro version 3.7.1 (Esri, Redlands, CA, USA) and the Model My Watershed web application within the WikiWatershed toolkit (Stroud Water Research Center). The contributing watershed upstream of each sampling site was delineated using the watershed-delineation function in Model My Watershed, and the resulting watershed boundaries were imported into ArcGIS Pro for further analysis. Site characteristics included elevation, mean watershed slope, total developed land, deciduous forest, cultivated cropland, stream density, Strahler stream order, and USDA Natural Resources Conservation Service hydrologic soil group. Stream order was determined for the channel at each sampling location as a hydrologic characteristic and was not used as a criterion for site selection.

Land-cover percentages were obtained from the 2019 National Land Cover Database (NLCD) available through Model My Watershed (Dewitz and U.S. Geological Survey, 2021). Total developed land was calculated as the sum of developed open-space and low-, medium-, and high-intensity developed land classes and expressed as a percentage of the contributing watershed area. Stream density was calculated as:


$$D\,s=\frac{L\,s}{A\,w}$$

where D_*s*_ is stream density (km km^–2^), L_*s*_ is the total stream length (km) within the contributing watershed, and A_*w*_ is the contributing watershed area (km^2^). Mean elevation and mean land-surface slope were calculated across each delineated contributing watershed. These variables were used to characterize spatial differences in urbanization and hydrologic conditions and were evaluated as potential explanatory variables for antibiotic resistance gene and microbial water quality marker composition.

During each sampling campaign, ambient air temperature, relative humidity, atmospheric pressure, water temperature, water depth, and precipitation were recorded to characterize field conditions. These measurements were used to document environmental conditions at the time of sampling and are summarized in Supplementary Table 1. To evaluate potential wet-weather conditions preceding sampling, antecedent precipitation was obtained from the NOAA/NCEI Detroit Metropolitan Airport station (GHCND: USW00094847). Cumulative precipitation was calculated for the 24-, 48-, and 72-h periods preceding each sampling event (Supplementary Table 2).

### 2.2 Water sampling and physicochemical analyses

Water samples were collected from six sites along the Rouge River watershed during one sampling campaign in each of three sampled years (2023, 2024, and 2026). Each campaign therefore represents a discrete snapshot of conditions across the watershed rather than continuous temporal or seasonal monitoring. At each site, three independent 1-L composite subsurface water samples were collected at a depth of approximately 30 cm below the water surface. Each composite sample consisted of four grab samples collected at 20-min intervals to minimize short-term temporal variability during the sampling period. Samples were collected in sterile glass containers, transported to the laboratory on ice, and processed within 8 h of collection.

Dissolved oxygen was measured in situ using a FiveGo portable dissolved oxygen meter (Mettler Toledo, Columbus, OH, USA). Three measurements were obtained at each site and averaged. In the laboratory, pH, electrical conductivity, total dissolved solids, and turbidity were measured in triplicate using a Hanna Edge HI2002 multiparameter meter, a Hanna HI9810 pH/EC/TDS meter, and a Hanna HI98703-01 portable turbidity meter, respectively (Hanna Instruments, Ann Arbor, MI, USA).

For molecular analyses, approximately 300–650 mL of each composite water sample was filtered independently through Sterivex™ filter units (Millipore, Billerica, MA, USA), with the final volume depending on filter clogging caused by suspended particulate matter. Filtration volume was therefore not standardized among samples and was not used to normalize the molecular data. This procedure yielded three Sterivex filters per sampling site. Filters were stored at −20 °C until DNA extraction. DNA extracted from the three filters collected at each sampling site was subsequently pooled for high-throughput qPCR array analysis, as described below.

### 2.3 DNA extraction

Genomic DNA was extracted independently from the three Sterivex™ filters collected at each sampling site using the DNeasy PowerWater Kit (Qiagen, Germantown, MD, USA) according to the manufacturer’s instructions. Briefly, filters were transferred to bead tubes for mechanical and chemical cell lysis, followed by inhibitor removal, DNA purification, and DNA elution. DNA concentration and purity were assessed using a NanoDrop™ 1000 spectrophotometer (Thermo Fisher Scientific, Waltham, MA, USA) and a Quantus™ Fluorometer (Promega Corporation, Madison, WI, USA). Equal volumes of DNA from the three biological replicate composite water samples were pooled to generate a single composite DNA sample for each site-year. Pooling provided sufficient DNA for the high-throughput qPCR arrays while limiting the number of arrays required for site-level molecular profiling. The resulting pooled DNA sample was treated as the analytical representation of each site-year sampling event. Consequently, qPCR-array analyses characterize the pooled site-year sample and do not quantify within-site variation among the three composite water samples. Composite DNA samples were stored at −20 °C until qPCR array analysis.

### 2.4 Quantitative PCR detection of microbial water quality markers and antibiotic resistance genes

Composite DNA samples were analyzed using the Microbial DNA Water Quality Array and the Microbial DNA Antibiotic Resistance Genes Array (Qiagen GmbH, Hilden, Germany). The Water Quality Array targets 50 microbial water quality markers associated with human, animal, and environmental fecal contamination, whereas the Antibiotic Resistance Genes Array targets 83 antibiotic resistance genes (ARGs) representing major antimicrobial resistance classes, including β-lactams, aminoglycosides, tetracyclines, macrolides, glycopeptides, sulfonamides, and multidrug resistance determinants. The commercial assays use target-specific PCR primer pairs and 5′-hydrolysis probes and were experimentally verified by the manufacturer for assay performance. According to QIAGEN, the Microbial DNA qPCR assays have primer efficiencies of 80%–120% and correlation coefficients (R) > 0.995 (QIAGEN, 2026). Amplicon size varies among individual target assays because each target is detected using a specific primer/probe set. Primer and probe sequences, primer lengths, and melting temperatures are not provided in the manufacturer’s publicly available specifications for these commercial arrays and therefore were not available for reporting. The individual microbial water quality markers included in the Water Quality Array are listed by functional category in Supplementary Table 3, whereas the ARG targets included in the Antibiotic Resistance Genes Array are identified by antibiotic resistance class in Figure 10.

For each array, 500 ng of pooled genomic DNA was combined with the Microbial DNA qPCR Master Mix according to the manufacturer’s instructions (QIAGEN, 2022) and dispensed into 96-well plates preloaded with gene-specific primers and hydrolysis probes. The standardized 500-ng DNA input controlled the amount of template DNA analyzed in each array but did not normalize targets to the volume of water filtered or account for differences in microbial biomass among samples. Accordingly, the qPCR results represent targeted DNA sequences within the standardized DNA mass analyzed rather than gene concentrations per unit volume of source water. Quantitative PCR was performed using a CFX Connect Real-Time PCR Detection System (Bio-Rad Laboratories, Hercules, CA, USA) under the following cycling conditions: 95 °C for 10 min, followed by 40 cycles of 95 °C for 15 s and 60 °C for 2 min (QIAGEN, 2022).

Raw amplification data were analyzed using the Qiagen GeneGlobe Data Analysis Center, which automatically assigned quantification cycle (Cq) values and quality-control flags according to the manufacturer’s analysis pipeline. Subsequent data processing included the classification of targets as positive, inconclusive, or not detected based on Cq thresholds, as described in the following section.

### 2.5 Molecular data processing and statistical analysis

#### 2.5.1 qPCR data processing

Quantification cycle (Cq) values generated by the Qiagen GeneGlobe Data Analysis Center (Qiagen GmbH, Hilden, Germany) were used to classify antibiotic resistance genes (ARGs) and microbial water quality markers as positive, inconclusive, or not detected. Following the manufacturer’s recommended criteria, targets with Cq values ≤ 35 were classified as positive, targets with Cq values > 35 and < 40 as inconclusive, and targets with Cq = 40 as not detected (QIAGEN, 2022). Target-specific standard curves, amplification efficiencies, and experimental limits of detection were not independently determined in this study; therefore, the Cq ≤ 35 criterion was treated as an operational detection threshold rather than as a target-specific, experimentally validated limit of detection.

Only positive detections (Cq ≤ 35) were included in detection-based analyses; targets with Cq values > 35 and < 40 were classified as inconclusive and were not treated as positive detections. Inconclusive and non-detected targets were excluded from these analyses but were retained in heat maps to provide a complete overview of target detection. A binary detection matrix based on positive detections (presence/absence) was used for all subsequent statistical and multivariate analyses. Detection-based analyses therefore reflect the occurrence of targeted DNA sequences under the specified Cq criterion and were not interpreted as absolute gene concentrations in the source water. Because the volume of water filtered and microbial biomass varied among samples, Cq values and detection-based metrics were interpreted within the standardized DNA input used for qPCR rather than as measures of target concentration per unit volume of source water. ARG richness and microbial marker richness were calculated as the total number of unique positively detected targets at each sampling site, whereas target detection frequency was defined as the number of sampling sites at which each target was positively detected.

#### 2.5.2 Classification of antibiotic resistance genes and microbial water quality markers

To facilitate interpretation of spatial patterns and differences among sampling campaigns, antibiotic resistance genes (ARGs) were grouped into their corresponding antimicrobial resistance classes (e.g., β-lactam, aminoglycoside, tetracycline, macrolide, sulfonamide, glycopeptide, and multidrug resistance) according to the Qiagen array annotation. Microbial water quality markers were similarly grouped into six functional categories based on their primary ecological or contamination-source association: (i) human fecal-associated markers, (ii) human-associated opportunistic pathogens, (iii) fecal indicator bacteria, (iv) animal-associated markers, (v) waterborne pathogens, and (vi) environmental source indicators. Marker classification was based on the annotations provided with the Qiagen Microbial DNA Water Quality Array and supplemented by published information on microbial ecology and host association. These functional categories were used to organize and summarize the microbial marker data and were not considered mutually exclusive, as individual microbial targets may have multiple ecological or source associations. These classifications were used to summarize detection patterns and compare the spatial distribution of ARGs and microbial water quality markers across the watershed. For comparisons among ARG resistance classes, detection frequency was normalized to the total number of targets represented within each resistance class to account for differences in array composition, as described in section “2.5.3 Data visualization.” The complete classification of microbial water quality markers is provided in Supplementary Table 3.

#### 2.5.3 Data visualization

Heat maps were generated to visualize the spatial distribution of positively detected antibiotic resistance genes (ARGs) and microbial water quality markers across the six sampling sites and sampling campaigns. Box-and-whisker plots were constructed using Cq values of positively detected targets (Cq ≤ 35) to compare Cq distributions among sampling sites and ARG resistance classes or microbial water quality marker functional categories. Because Cq values were not normalized to a reference gene, converted to absolute gene copy numbers, or normalized to the volume of source water filtered, the box plots were used to compare Cq distributions among positively detected targets and were not interpreted as measures of relative or absolute environmental abundance.

To compare detection among ARG resistance classes and microbial water quality marker functional categories that contained different numbers of targets on the arrays, class-normalized detection frequency was calculated for each sampling site and year as:


N⁢D⁢F⁢c,s,y=D,c,s,yN⁢c⁢x⁢ 100

where NDF_*c,s,y*_ is the class-normalized detection frequency (%) of class or category *c* at site *s* in year *y*, D_*c,s,y*_ is the number of positively detected targets belonging to that class or category at the corresponding site-year, and N_*c*_ is the total number of targets represented in that class or category on the respective qPCR array.

Relative composition was calculated as the percentage contribution of each ARG resistance class or microbial water quality marker functional category to the total number of positively detected targets within each site-year:


R⁢C⁢c,s,y=D,c,s,yD,s,y⁢x⁢ 100

where RC_*c,s,y*_ is the relative composition (%) of class or category *c* at site *s* in year *y*, D_*c,s,y*_ is the number of positively detected targets belonging to that class or category, and D_*s,y*_ is the total number of positively detected targets across all ARG resistance classes or microbial water quality marker categories at that site-year. Bar charts were generated to summarize class-normalized detection frequency and relative composition across sampling sites and sampling campaigns.

#### 2.5.4 Statistical analysis

Physicochemical variables were analyzed using two-way analysis of variance (ANOVA) with sampling site and sampling year as fixed factors, including their interaction. When significant effects were detected, pairwise comparisons were performed using Tukey’s honestly significant difference (HSD) test. Model assumptions were evaluated using residual diagnostics, and data were transformed when necessary. Descriptive statistics were calculated for all measured variables.

Pearson correlation analysis was performed to explore linear relationships among watershed characteristics, physicochemical water quality variables, total animal abundance, antibiotic resistance gene (ARG) richness, and microbial water quality marker richness. Pearson correlation coefficients (r) were calculated using pooled site-year observations. Watershed characteristics and total animal abundance were site-level variables that remained constant across sampling years and were therefore repeated for the corresponding site-year observations. Because these repeated values do not represent independent temporal measurements, correlations involving these variables were treated as exploratory and interpreted as associations rather than independent temporal relationships. Results of the Pearson correlation analyses are presented in Supplementary Table 4.

#### 2.5.5 Multivariate ordination analysis

Multivariate ordination analyses were performed separately for the antibiotic resistance gene (ARG) and microbial water quality marker datasets to evaluate compositional variation among sampling sites and sampling campaigns and to assess relationships with environmental variables. Binary detection matrices were constructed by coding positively detected targets as 1 and inconclusive or non-detected targets as 0. Non-metric multidimensional scaling (NMDS) based on Jaccard dissimilarity was used as the primary unconstrained ordination to visualize differences in target composition among samples, whereas principal coordinates analysis (PCoA), also based on Jaccard dissimilarity, was performed as a complementary unconstrained ordination.

Relationships between ARG or microbial water quality marker composition and environmental variables were evaluated using redundancy analysis (RDA) and canonical correspondence analysis (CCA). Prior to RDA, binary target-detection matrices were Hellinger transformed to reduce the influence of dominant targets and make the data suitable for Euclidean-based ordination. CCA was performed using chi-square distances to evaluate relationships between target composition and environmental variables for comparison with the RDA results. Constrained ordination models included percentage developed land, stream density, conductivity, dissolved oxygen, pH, turbidity, and total animal abundance as explanatory variables. Percentage forest, percentage cropland, and total dissolved solids were excluded because of collinearity with developed land and conductivity, respectively.

The ordination dataset comprised 18 observations representing six sites sampled during three sampling campaigns. Geographic information system (GIS)-derived watershed characteristics were based on a single set of spatial data and therefore did not vary among the 2023, 2024, and 2026 campaigns; the same site-specific values were assigned to each site’s three observations. In contrast, physicochemical variables were measured during each sampling campaign and varied among observations. The GIS variables represented fixed spatial characteristics rather than independent temporal measurements. To account for the repeated observations within sites, statistical significance of the RDA was evaluated by permuting the six site blocks while preserving the three sampling-campaign observations within each site (720 possible site-block permutations). Significance of the constrained RDA axes was evaluated using the same site-block permutation structure. Environmental vectors in the RDA ordination were interpreted descriptively as the direction and relative strength of associations with target composition rather than as evidence of statistically significant individual effects.

The significance of the overall CCA model was evaluated using 999 permutations. NMDS and RDA were selected as the primary ordination methods because they provided complementary unconstrained and constrained analyses of the binary detection data. PCoA and CCA were performed as complementary analyses and are presented in Supplementary Figures 1, 2.

#### 2.5.6 Software

Heat maps, box-and-whisker plots, bar charts, and hierarchical clustering heat maps were generated using OriginPro 2026b (OriginLab Corporation, Northampton, MA, USA). All statistical analyses described above were performed in R version 4.5.1 (R Foundation for Statistical Computing, Vienna, Austria).

## 3 Results

### 3.1 GIS characterization of the urbanization gradient

GIS analyses confirmed a pronounced urbanization gradient across the six Rouge River sampling sites (Figures 1, 2 and Table 1). Total developed land increased from 46.3% at the upstream Canton site to 92.7% at the downstream Rouge Park site, accompanied by progressive declines in deciduous forest (16.5%–3.4%) and cultivated cropland (25.2%–0.01%). Venoy, Inkster, Ford Field, and Dearborn had intermediate proportions of developed land, ranging from 61.0% to 67.5%, while site-specific land-cover maps showed differences in land-cover composition among the contributing watersheds and a general transition from more agricultural and forested upstream landscapes to highly developed downstream landscapes (Figure 2). Hydrologic characteristics also varied along the watershed gradient, with stream order increasing from third order at Canton to fifth order at Rouge Park. Stream density ranged from 0.787 to 1.090 km km^–2^, with the highest values at Venoy and Inkster, whereas elevation (214.0–234.4 m) and mean watershed slope (0.8%–1.9%) varied modestly among sites. Upstream and midstream sites were primarily associated with USDA Hydrologic Soil Group C/D, whereas the downstream sites (Ford Field, Dearborn, and Rouge Park) were classified as Group C soils. Together, these landscape and hydrologic characteristics characterized the environmental gradient across the six Rouge River sampling sites.

**FIGURE 2:**
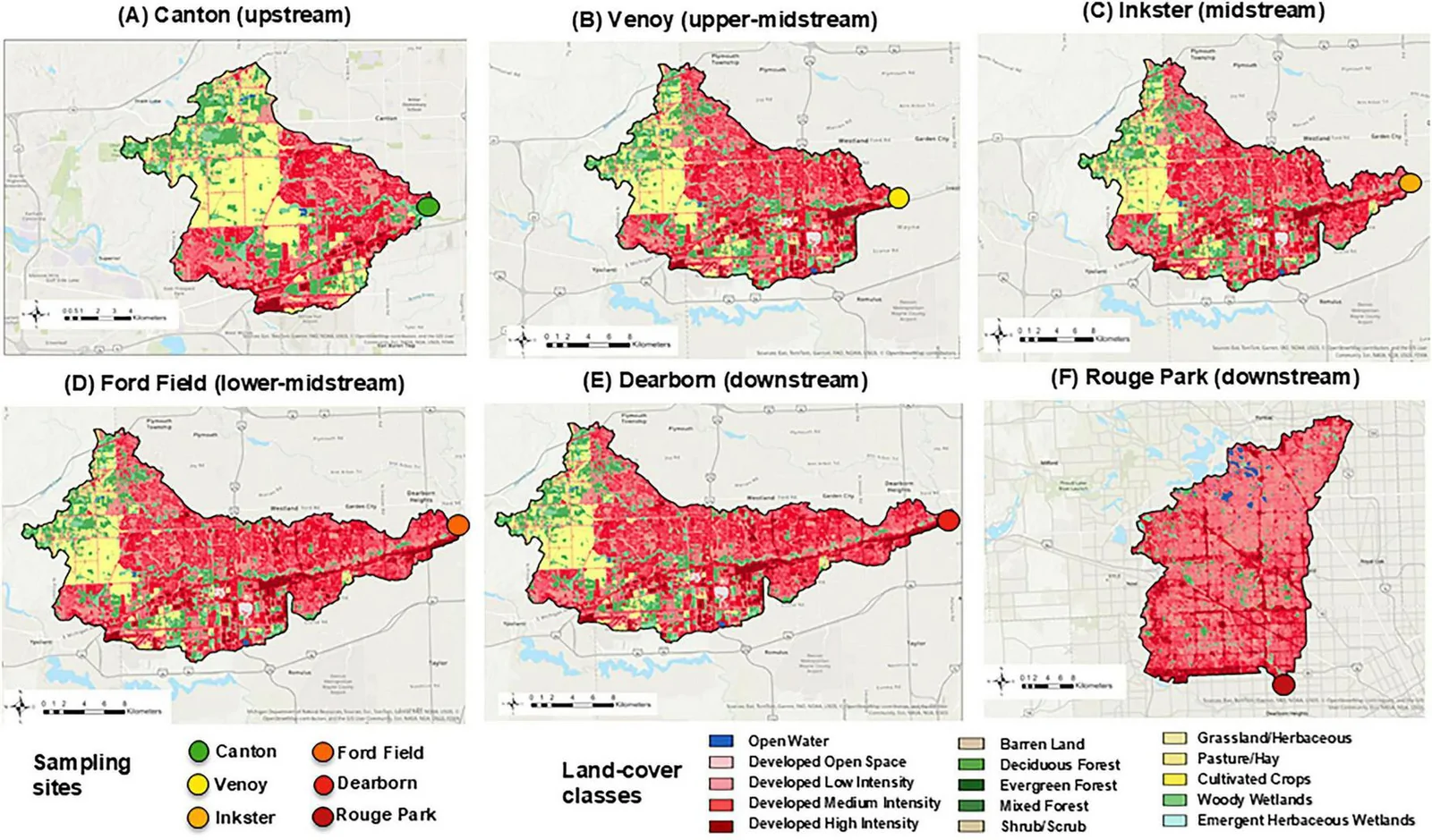
Six-panel GIS map showing the contributing watersheds for the six Rouge River sampling locations: **(A)** Canton (upstream), **(B)** Venoy (upper-midstream), **(C)** Inkster (midstream), **(D)** Ford Field (lower-midstream), **(E)** Dearborn (downstream), and **(F)** Rouge Park (downstream). Each panel shows the delineated watershed boundary, sampling location, and color-coded National Land Cover Database (NLCD) land-cover classes. The maps illustrate differences in land-cover composition among the contributing watersheds, with developed land generally becoming more prominent toward the downstream sampling locations. Land-cover composition of the contributing watersheds associated with the six Rouge River sampling locations used in the geospatial analyses. GIS maps generated using ArcGIS Pro illustrate the delineated contributing watershed for **(A)** Canton (upstream), **(B)** Venoy (upper-midstream), **(C)** Inkster (midstream), **(D)** Ford Field (lower-midstream), **(E)** Dearborn (downstream), and **(F)** Rouge Park (downstream). Land-cover classes represent National Land Cover Database (NLCD) categories, including developed land, forest, agricultural land, wetlands, open water, and other land-cover types used to characterize watershed land use.

### 3.2 Environmental characteristics

Physicochemical characteristics exhibited pronounced variation among sampling sites and campaigns across the Rouge River watershed (Table 2 and Supplementary Table 5). Mean water temperature was highest during the 2024 sampling period across all sites and lowest in 2026, whereas dissolved oxygen differed markedly among sampling campaigns, with generally lower concentrations in 2024 and higher concentrations in 2026, particularly at Ford Field and Rouge Park. River water remained circumneutral throughout the study, with pH values ranging from 6.08 to 7.55. Electrical conductivity and total dissolved solids (TDS) followed similar spatial patterns, with consistently higher values at the downstream sites, particularly Ford Field, Dearborn, and Rouge Park. Turbidity also varied among sites and sampling campaigns and was generally highest at Rouge Park during 2023 and 2024. Antecedent precipitation varied among sampling events, with 72-h cumulative precipitation ranging from 0 to 37.34 mm (Supplementary Table 2). At Inkster, 72-h antecedent precipitation was 20.83, 4.83, and 9.40 mm in 2023, 2024, and 2026, respectively, indicating contrasting antecedent rainfall conditions among sampling campaigns.

**TABLE 2:** Physicochemical characteristics of river water at six sampling sites in the Rouge River watershed during 2023, 2024, and 2026.

Site	Year	Water temperature (°C)	Dissolved oxygen (mg/L)	pH	Conductivity (μ S/cm)	TDS (mg/L)	Turbidity (NTU)
Canton	2023	19.50 ± 0.10	10.58 ± 0.28	6.63 ± 0.14	1053.33 ± 40.41	526.67 ± 28.87	20.07 ± 1.35
	2024	22.23 ± 0.40	2.64 ± 0.08	6.93 ± 0.06	343.33 ± 5.77	198.67 ± 5.77	45.801 ± 12.97
	2026	18.37 ± 0.29	12.86 ± 0.91	6.12 ± 0.10	689.67 ± 20.87	354.33 ± 5.77	46.10 ± 0.75
Venoy	2023	19.33 ± 0.15	9.19 ± 0.40	6.98 ± 0.47	1026.67 ± 30.55	526.67 ± 28.87	20.07 ± 1.35
	2024	24.47 ± 0.40	9.19 ± 0.40	6.44 ± 0.11	263.33 ± 25.17	165.33 ± 5.77	19.60 ± 1.49
	2026	15.87 ± 0.31	12.05 ± 1.74	6.13 ± 0.11	1093.03 ± 20.00	554.33 ± 5.77	25.57 ± 1.45
Inkster	2023	18.90 ± 0.10	11.37 ± 0.27	7.0 ± 0.13	494.33 ± 55.08	244.00 ± 39.34	32.53 ± 5.43
	2024	24.40 ± 0.17	3.02 ± 0.10	6.84 ± 0.17	336.67 ± 15.28	204.67 ± 14.19	21.13 ± 5.08
	2026	23.33 ± 0.47	9.38 ± 1.14	6.08 ± 0.08	906.33 ± 55.08	461.00 ± 26.46	30.20 ± 1.11
Ford Field	2023	17.07 ± 0.21	8.58 ± 0.05	6.66 ± 0.10	1263.33 ± 5.77	640.00 ± 10.00	17.87 ± 0.91
	2024	21.13 ± 0.12	3.50 ± 0.31	7.11 ± 0.13	426.67 ± 11.55	238.67 ± 11.55	10.99 ± 2.11
	2026	13.10 ± 0.53	14.60 ± 3.17	6.28 ± 0.03	1509.67 ± 20.82	761.00 ± 10.00	10.57 ± 0.35
Dearborn	2023	19.47 ± 0.15	3.70 ± 0.18	6.99 ± 0.28	1616.67 ± 290.23	750.00 ± 45.83	11.07 ± 0.38
	2024	24.47 ± 0.67	3.82 ± 0.22	6.48 ± 0.19	275.33 ± 5.77	496.67 ± 5.77	35.05 ± 0.35
	2026	14.27 ± 0.50	7.60 ± 0.62	7.05 ± 0.03	1399.67 ± 25.17	721.00 ± 0.00	9.56 ± 1.79
Rouge Park	2023	19.03 ± 0.21	6.40 ± 0.10	7.1 ± 0.14	790.00 ± 7.94	553.00 ± 10.60	56.43 ± 10.60
	2024	20.67 ± 0.38	1.76 ± 0.06	7.55 ± 0.18	520.00 ± 182.48	316.67 ± 5.77	48.87 ± 15.52
	2026	12.30 ± 0.36	14.16 ± 0.48	6.38 ± 0.03	2253.00 ± 55.68	1124.33 ± 0.51	11.63 ± 0.51
2-way ANOVA results							
Site	–	***	***	***	***	***	***
Year	–	***	***	***	***	***	**
Site × Year	–	***	***	***	***	***	***

ns, *P* ≥ 0.05;

*, *P* < 0.05;

**, *P* < 0.01;

***, *P* < 0.001.

Two-way ANOVA identified significant effects of sampling site, sampling year, and their interaction for all measured physicochemical variables (Supplementary Table 5). Water temperature, dissolved oxygen, pH, electrical conductivity, and TDS each exhibited significant Site, Year, and Site × Year effects (*P* < 0.001). Turbidity likewise differed significantly among sites (*P* < 0.001), years (*P* = 0.004), and Site × Year interactions (*P* < 0.001). The significant Site effects indicate that physicochemical conditions differed spatially across the watershed, whereas the significant Year effects indicate differences among the three sampling campaigns. The significant Site × Year interactions further indicate that the magnitude or direction of differences among sites varied among sampling campaigns. Together, these results demonstrate that environmental conditions across the Rouge River watershed were spatially heterogeneous and differed substantially among the three sampling campaigns.

### 3.3 Microbial water quality markers

#### 3.3.1 Detection frequency and relative composition of microbial water quality marker categories

The detection frequency and relative composition of microbial water quality marker categories varied among sampling sites and sampling campaigns (Figures 3, 4). All six functional categories were detected across the Rouge River watershed, although their relative representation differed among sites and sampling campaigns. Waterborne pathogens and animal-associated markers were consistently detected and accounted for the largest proportions of positively detected microbial markers (Cq ≤ 35) at most sampling sites. In contrast, human-associated opportunistic pathogens, environmental source indicators, human fecal-associated markers, and fecal indicator bacteria generally contributed smaller proportions of the detected microbial marker profile.

**FIGURE 3:**
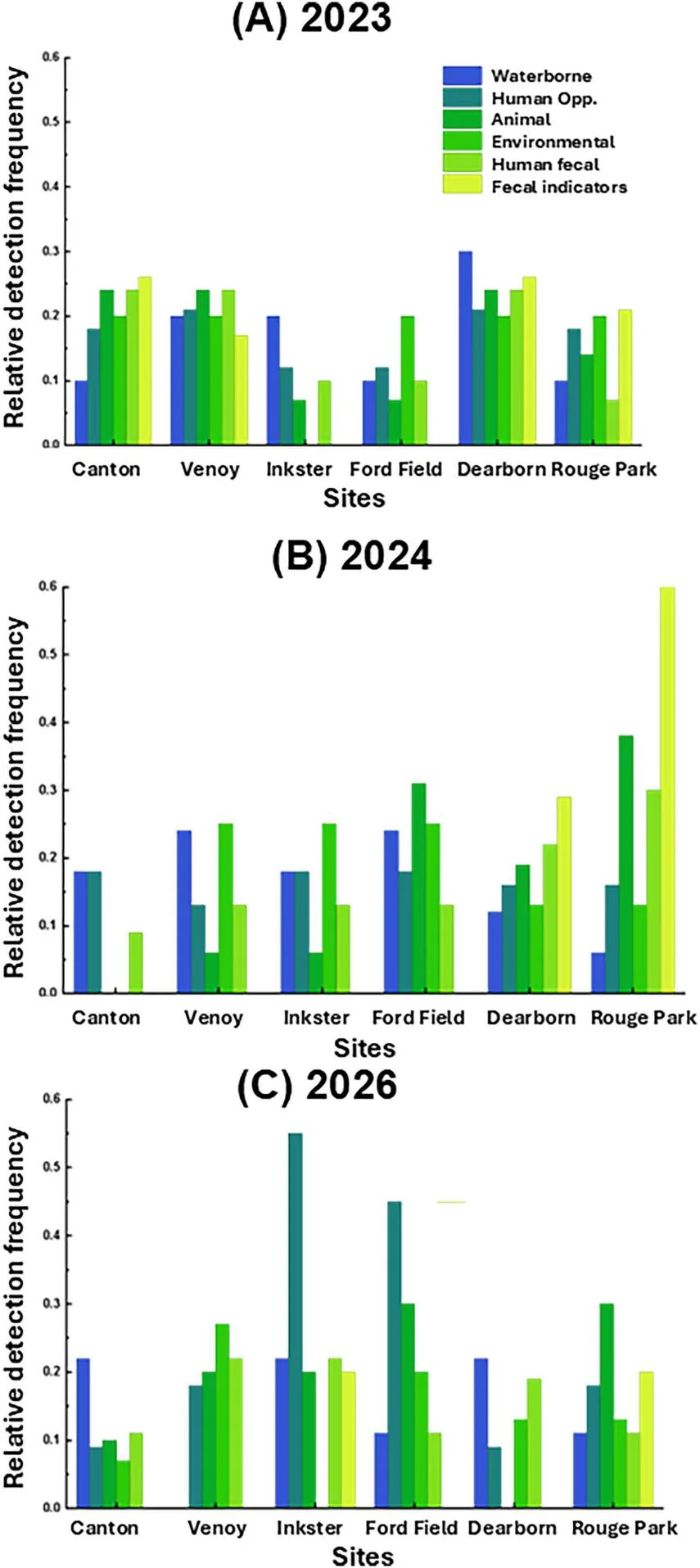
Three grouped bar charts showing the class-normalized detection frequency of six microbial water quality marker functional categories across six Rouge River sampling sites during 2023, 2024, and 2026. The x-axis shows sampling sites (Canton, Venoy, Inkster, Ford Field, Dearborn, and Rouge Park), and the y-axis shows class-normalized detection frequency. Colored bars represent six microbial water quality marker functional categories: waterborne pathogens, human-associated opportunistic pathogens, animal-associated markers, environmental source indicators, human fecal-associated markers, and fecal indicator bacteria. Class-normalized detection frequency of microbial water quality marker functional categories across six Rouge River sampling sites during **(A)** 2023, **(B)** 2024, and **(C)** 2026. Bars represent the proportion of positively detected microbial water quality markers (Cq ≤ 35) within each functional category at each sampling site. Class-normalized detection frequency was calculated as the proportion of positively detected markers within each functional category relative to the total number of markers represented in that category. Functional categories include waterborne pathogens, human-associated opportunistic pathogens, animal-associated markers, environmental source indicators, human fecal-associated markers, and fecal indicator bacteria. The complete classification of microbial water quality markers is provided in Supplementary Table 3.

**FIGURE 4:**
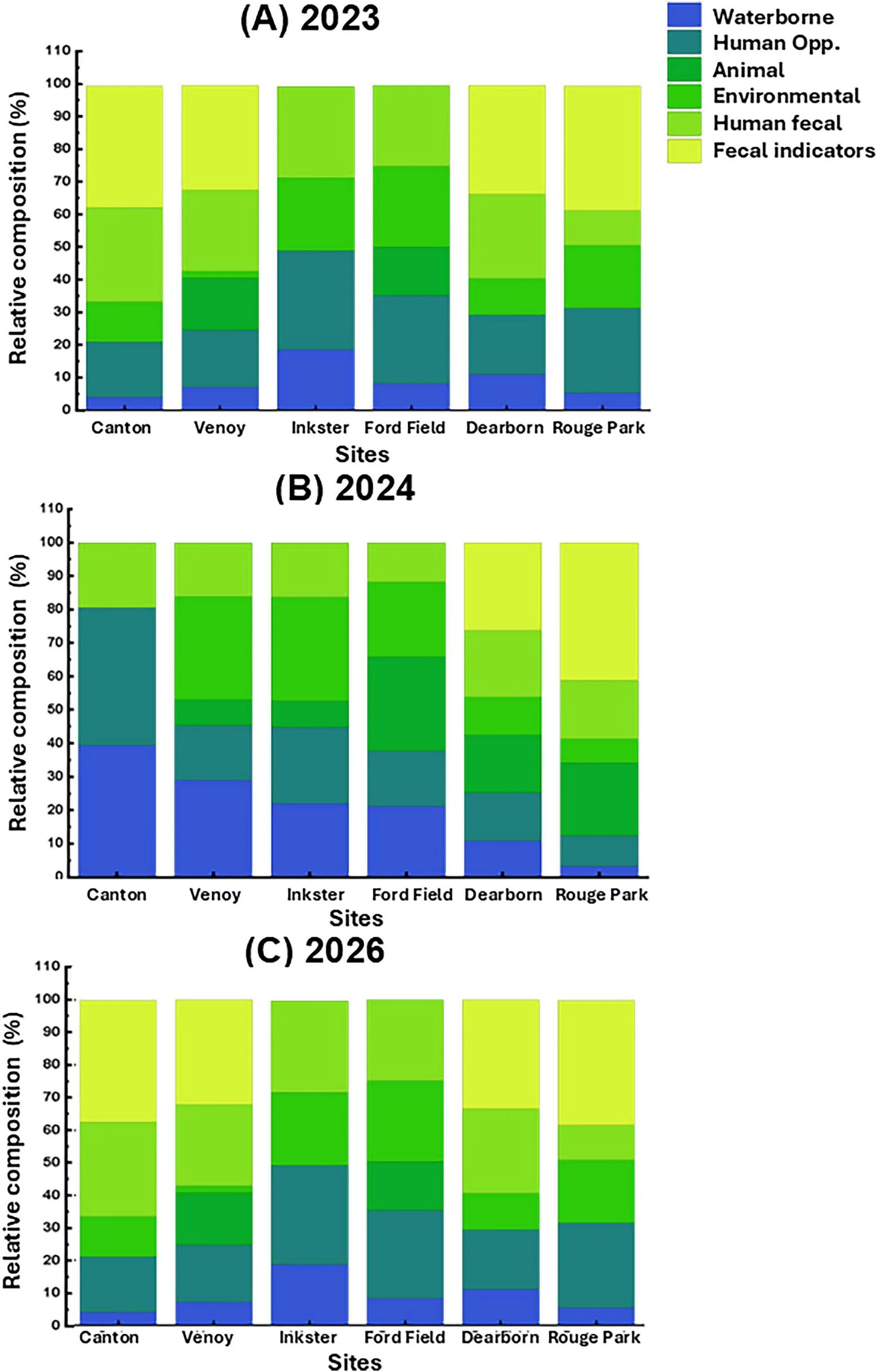
Three stacked bar charts showing the relative composition of microbial water quality marker functional categories across six Rouge River sampling sites during 2023, 2024, and 2026. Each stacked bar represents one sampling site and is divided into colored segments corresponding to six microbial water quality marker functional categories. The y-axis shows the relative composition (%) of positively detected microbial water quality markers. Relative composition of microbial water quality marker functional categories across six Rouge River sampling sites during **(A)** 2023, **(B)** 2024, and **(C)** 2026. Stacked bars show the percentage contribution of each functional category to the total number of positively detected microbial water quality markers (Cq ≤ 35) at each sampling site. Functional categories are indicated in the legend, and the complete classification of microbial water quality markers is provided in Supplementary Table 3.

Differences were evident among the three sampling campaigns. In 2023, both detection frequency and marker composition were relatively similar among sampling sites, although Rouge Park exhibited a greater contribution of animal-associated markers and fecal indicator bacteria. Greater spatial variation emerged in 2024, with Ford Field showing increased detection and relative representation of environmental source indicators, whereas Rouge Park was characterized by a higher proportion of animal-associated markers and fecal indicator bacteria. A similar pattern was observed in 2026, when environmental source indicators remained prominent at Venoy and Ford Field, while animal-associated markers continued to dominate at Inkster and Rouge Park. Overall, both detection frequency and relative composition revealed consistent occurrences of all microbial water quality marker categories but site- and campaign-specific differences in their relative representation across the Rouge River watershed.

#### 3.3.2 Spatial distribution of individual microbial water quality markers

Heat maps generated for each sampling campaign revealed pronounced variation among sampling sites and campaigns in the distribution of individual microbial water quality markers across the six Rouge River sampling sites (Figure 5). Although many of the 50 microbial markers were not positively detected (Cq ≤ 35), several occurred consistently across multiple sampling sites and sampling campaigns. Waterborne pathogens were the most frequently detected functional group, with *Aeromonas* spp. (*A. enteropelogenes*, *A. punctata*, and *A. media*) and *Arcobacter butzleri* exhibiting widespread distribution throughout the watershed. In contrast, environmental source indicators, including *Desulfovibrio* spp., *Legionella pneumophila*, and *Mycobacterium* spp., were detected infrequently or were absent from most sampling sites.

**FIGURE 5:**
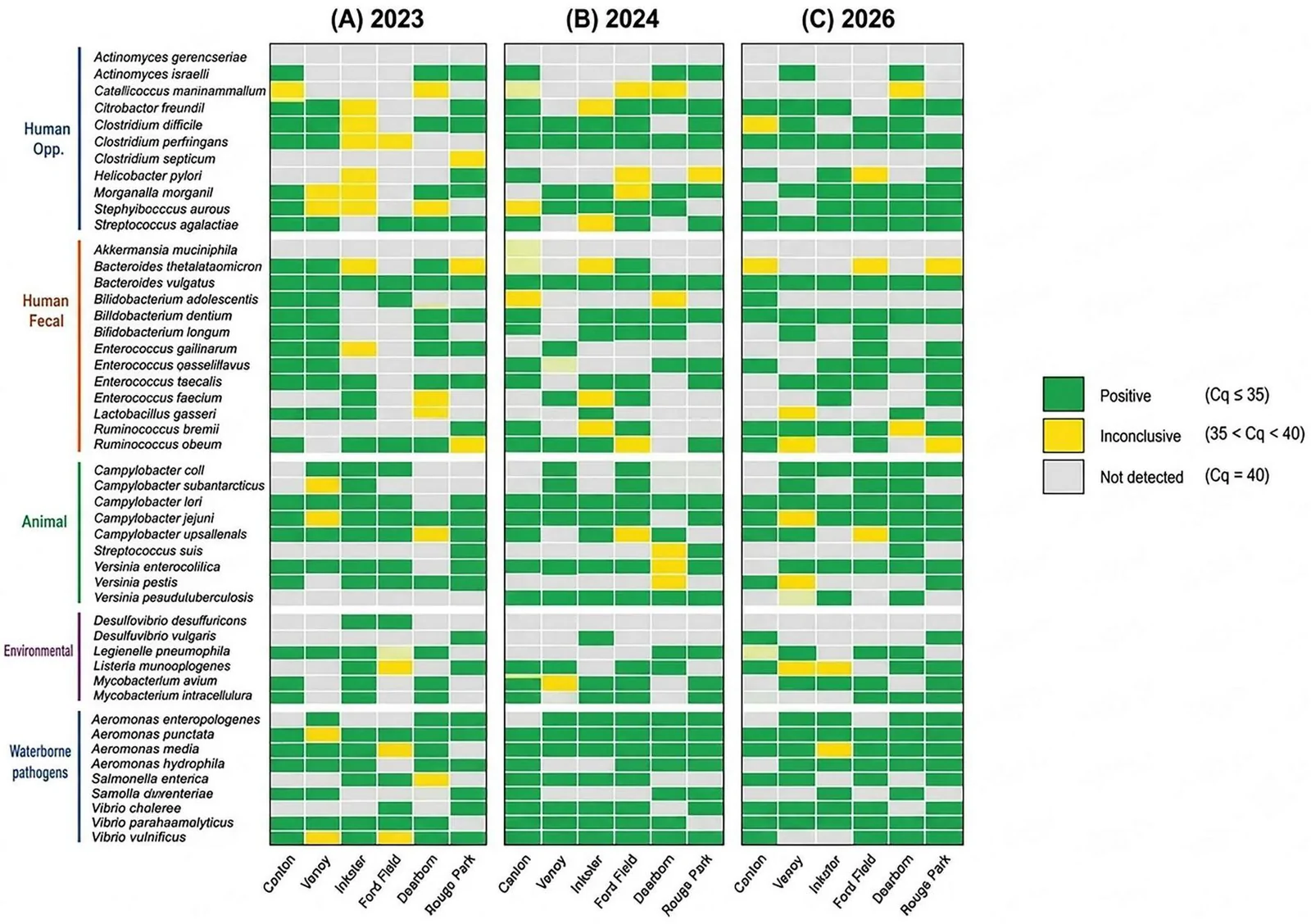
Three heat maps showing qPCR detection of 50 microbial water quality markers across six Rouge River sampling sites during 2023, 2024, and 2026. Rows represent individual microbial water quality markers grouped into six functional categories, and columns represent sampling sites arranged from upstream (Canton) to downstream (Rouge Park). Green cells indicate positive detection (Cq ≤ 35), yellow cells indicate inconclusive detection (Cq > 35 and < 40), and light gray cells indicate no detection (Cq = 40). Spatial distribution of microbial water quality markers across six Rouge River sampling sites during **(A)** 2023, **(B**) 2024, and **(C)** 2026. Heat maps summarize qPCR detection of 50 microbial water quality markers grouped into six functional categories. Rows represent individual microbial water quality markers, and columns represent sampling sites arranged from upstream (Canton) to downstream (Rouge Park). Green indicates positive detection (Cq ≤ 35), yellow indicates inconclusive detection (Cq > 35 and < 40), and light gray indicates no detection (Cq = 40).

Among the remaining functional groups, *Akkermansia muciniphila* and *Bacteroides vulgatus* were the most consistently detected human fecal-associated markers, whereas human-associated opportunistic pathogens occurred sporadically across the watershed. Animal-associated markers were dominated by *Campylobacter* spp., while *Yersinia* spp. and *Streptococcus suis* were detected less frequently.

Differences were also evident among the three sampling campaigns. In 2023, waterborne pathogens and selected human- and animal-associated markers were broadly distributed across the watershed. In 2024, fewer positive detections and more inconclusive results were observed for several microbial markers. In 2026, waterborne pathogens remained widespread, whereas human- and animal-associated markers exhibited greater site specificity. Collectively, the heat maps demonstrate that individual microbial water quality marker profiles varied considerably across sampling sites and sampling campaigns despite the persistent occurrence of several widespread waterborne pathogens.

#### 3.3.3 Cq distributions of positively detected microbial water quality markers

The distribution of Cq values for positively detected microbial water quality markers (Cq ≤ 35) varied among sampling sites, functional categories, and sampling campaigns (Figure 6). Median Cq values were generally similar across sampling sites, although Inkster exhibited greater variability in 2023 and 2024. Human-associated opportunistic pathogens and waterborne pathogens displayed broader Cq distributions than the remaining functional categories, whereas human fecal-associated markers and fecal indicator bacteria exhibited comparatively narrow distributions. Most positively detected markers had Cq values between approximately 28 and 35 across all three sampling campaigns.

**FIGURE 6:**
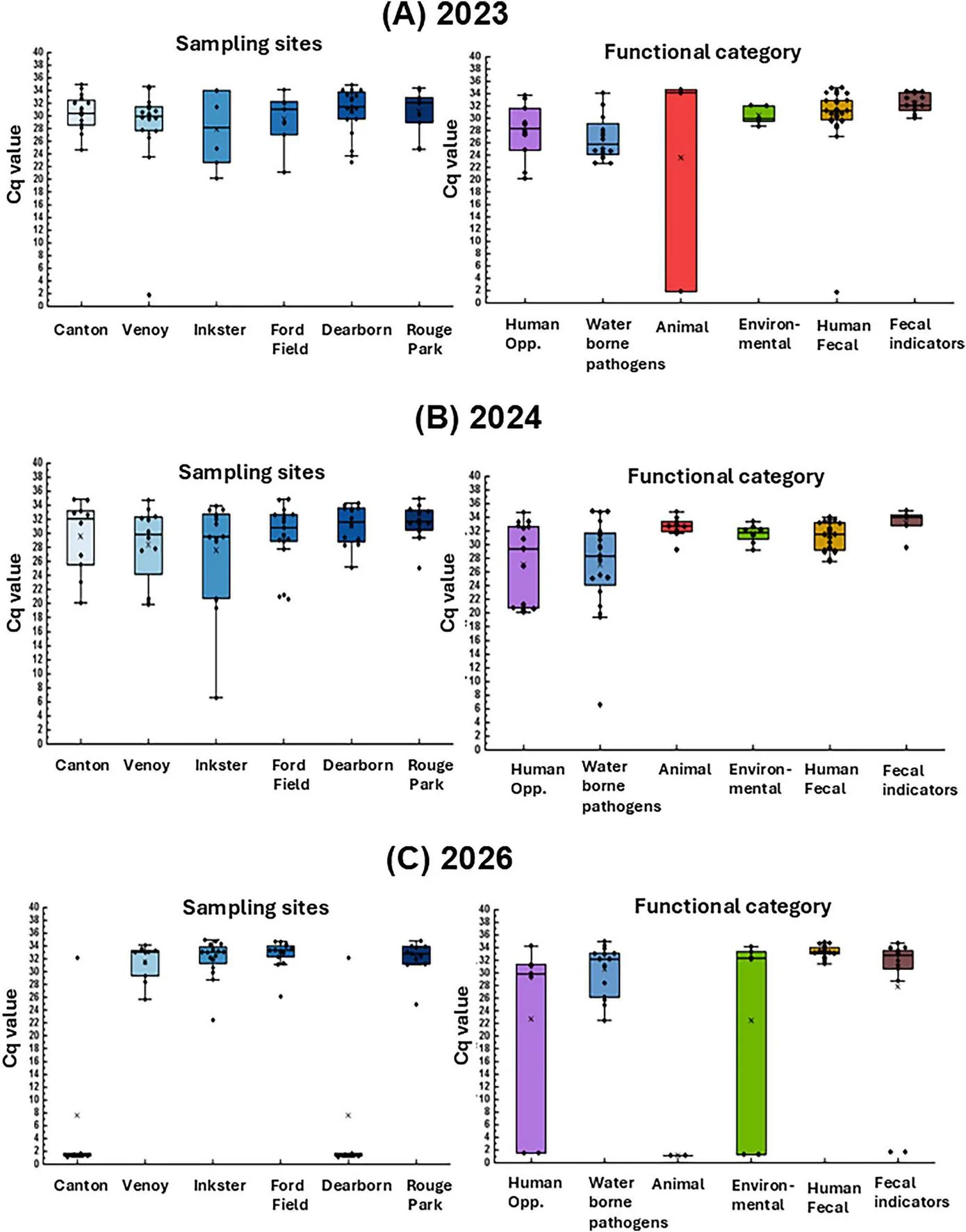
Six box-and-whisker plots arranged in three rows for 2023, 2024, and 2026. Left panels compare qPCR Cq values of positively detected microbial water quality markers (Cq ≤ 35) among six Rouge River sampling sites, and right panels compare Cq values among six microbial water quality marker functional categories. The plots show the distribution and variability of Cq values among sites and functional categories for each sampling campaign. Distribution of qPCR Cq values for positively detected microbial water quality markers across sampling sites and functional categories during **(A)** 2023, **(B)** 2024, and **(C)** 2026. Box-and-whisker plots summarize Cq values for positively detected microbial water quality markers (Cq ≤ 35). Left panels compare sampling sites, and right panels compare microbial water quality marker functional categories. Cq values represent amplification characteristics of positively detected targets within the standardized DNA input and are not interpreted as measures of relative or absolute environmental abundance.

#### 3.3.4 Hierarchical clustering of microbial water quality marker profiles

Hierarchical clustering revealed sampling campaign-specific similarities among sampling sites and microbial water quality marker functional categories (Figure 7). Clustering patterns differed among the three sampling campaigns, indicating differences in the relationships among sampling sites across the sampled events. In 2023, Canton clustered most closely with Rouge Park, whereas Dearborn and Inkster formed a separate subgroup. In 2024, Venoy and Inkster exhibited the greatest similarity, while Ford Field and Rouge Park formed a second cluster. In 2026, Ford Field clustered with Canton, whereas Inkster and Dearborn grouped together before clustering with Rouge Park. Functional categories also exhibited differences among sampling campaigns, with human-associated opportunistic pathogens clustering with environmental source indicators in 2023, fecal indicator bacteria clustering with human fecal-associated markers in 2024, and animal-associated markers clustering with fecal indicator bacteria in 2026. These hierarchical clustering results demonstrate that relationships among sampling sites and microbial water quality marker profiles differed among the three sampling campaigns.

**FIGURE 7:**
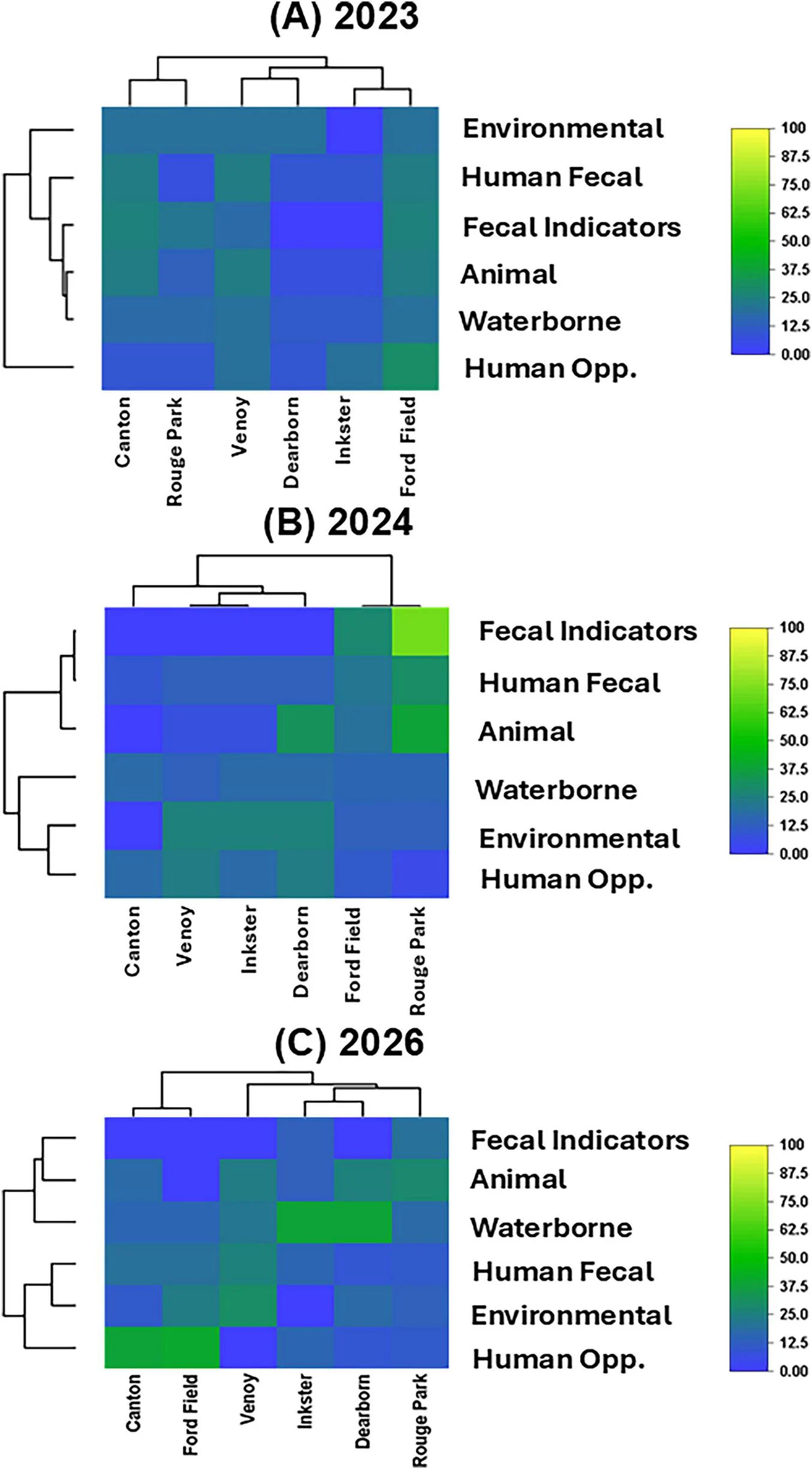
Three clustered heat maps showing the class-normalized detection frequency of six microbial water quality marker functional categories across six Rouge River sampling sites during 2023, 2024, and 2026. Rows represent microbial water quality marker functional categories, columns represent sampling sites, and dendrograms indicate hierarchical clustering of similar detection patterns. Cell color ranges from blue (low class-normalized detection frequency) to yellow (high class-normalized detection frequency). Hierarchical clustering heat maps of microbial water quality marker functional categories across six Rouge River sampling sites during **(A)** 2023, **(B)** 2024, and **(C)** 2026. Heat maps show the class-normalized detection frequency (%) of positively detected (Cq ≤ 35) microbial water quality markers within six functional categories across the six sampling sites. Rows represent microbial water quality marker functional categories, columns represent sampling sites, and dendrograms illustrate hierarchical clustering based on similarities in detection patterns. Color intensity corresponds to class-normalized detection frequency, ranging from low (blue) to high (yellow).

#### 3.3.5 Multivariate ordination of microbial water quality marker profiles

Multivariate ordination analyses revealed variation in microbial water quality marker composition among sampling sites and sampling campaigns but no significant relationships with the measured watershed and physicochemical variables (Figure 8). NMDS indicated gradual compositional turnover among samples, with substantial overlap and no distinct clustering by sampling site or sampling campaign (stress = 0.062; Figure 8A). This overlap indicates that differences in microbial marker composition were not organized into consistent site- or sampling campaign-specific groupings across the watershed. Similarly, RDA explained 41.6% of the variation in microbial marker composition, but the overall model was not statistically significant (adjusted R^2^ = 0.008, permutation test, *P* = 0.491; Figure 8B), indicating that the measured environmental variables did not significantly account for the observed compositional variation.

**FIGURE 8:**
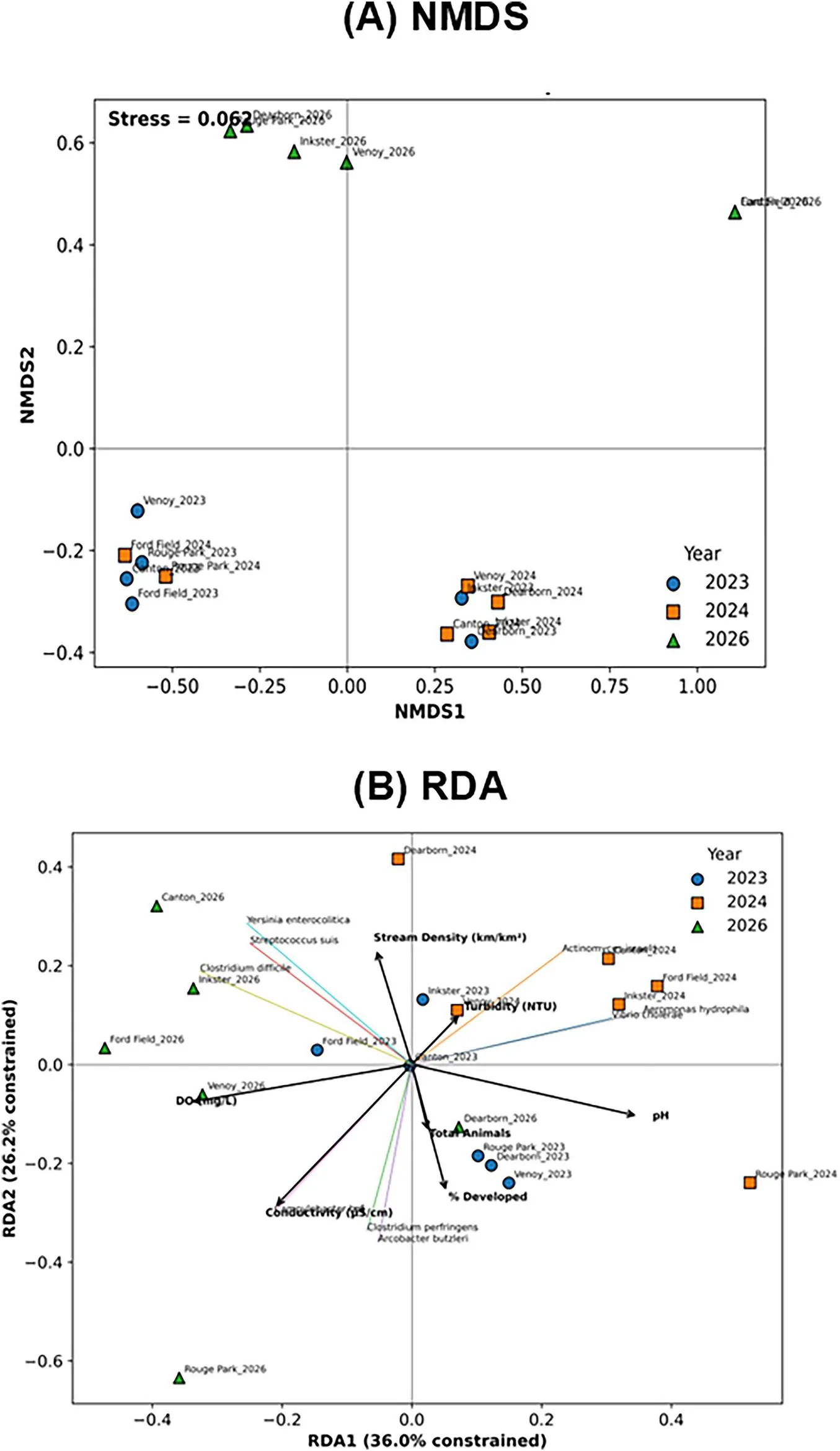
Two-panel ordination Figure showing microbial water quality marker composition across six Rouge River sampling sites sampled in 2023, 2024, and 2026. **(A)** Shows a non-metric multidimensional scaling (NMDS) ordination based on Jaccard dissimilarity with individual site-year samples. **(B)** Shows a redundancy analysis (RDA) ordination of Hellinger-transformed microbial water quality marker detection data with arrows representing selected watershed characteristics and physicochemical variables associated with sample composition. Multivariate ordination of microbial water quality marker composition across six Rouge River sampling sites during 2023, 2024, and 2026. **(A)** Non-metric multidimensional scaling (NMDS) ordination based on Jaccard dissimilarity showing similarities in microbial water quality marker composition among sampling sites and years (stress = 0.062). **(B)** Redundancy analysis (RDA) of Hellinger-transformed microbial water quality marker detection data showing relationships between microbial water quality marker composition and selected watershed characteristics and physicochemical variables. Arrows represent environmental variables, with vector direction indicating the direction of association and vector length indicating the relative strength of the relationship.

Supporting ordination analyses showed patterns consistent with the primary analyses (Supplementary Figure 1). PCoA showed a sample distribution similar to that observed with NMDS, while CCA was likewise not statistically significant (*P* = 0.401; Supplementary Figure 1). Collectively, these analyses indicate that although microbial water quality marker composition varied among samples, this variation was not systematically associated with the measured watershed or physicochemical gradients.

### 3.4 Antibiotic resistance genes

#### 3.4.1 Detection frequency and class distribution of antibiotic resistance genes

The number and class distribution of positively detected antibiotic resistance genes (ARGs; Cq ≤ 35) differed across locations and sampling campaigns (Figure 9). In 2023, Venoy had the greatest ARG richness (25 genes), whereas Inkster had the fewest detected ARGs (nine genes). This pattern shifted in 2024, when Inkster contained the greatest ARG richness (31 genes) and Rouge Park the fewest (10 genes). A similar pattern occurred in 2026: Inkster again had the highest ARG richness (31 genes), while Rouge Park remained the site with the fewest detected ARGs (10 genes). Canton and Ford Field each contained 21 detected ARGs, whereas Venoy and Dearborn each contained 18.

**FIGURE 9:**
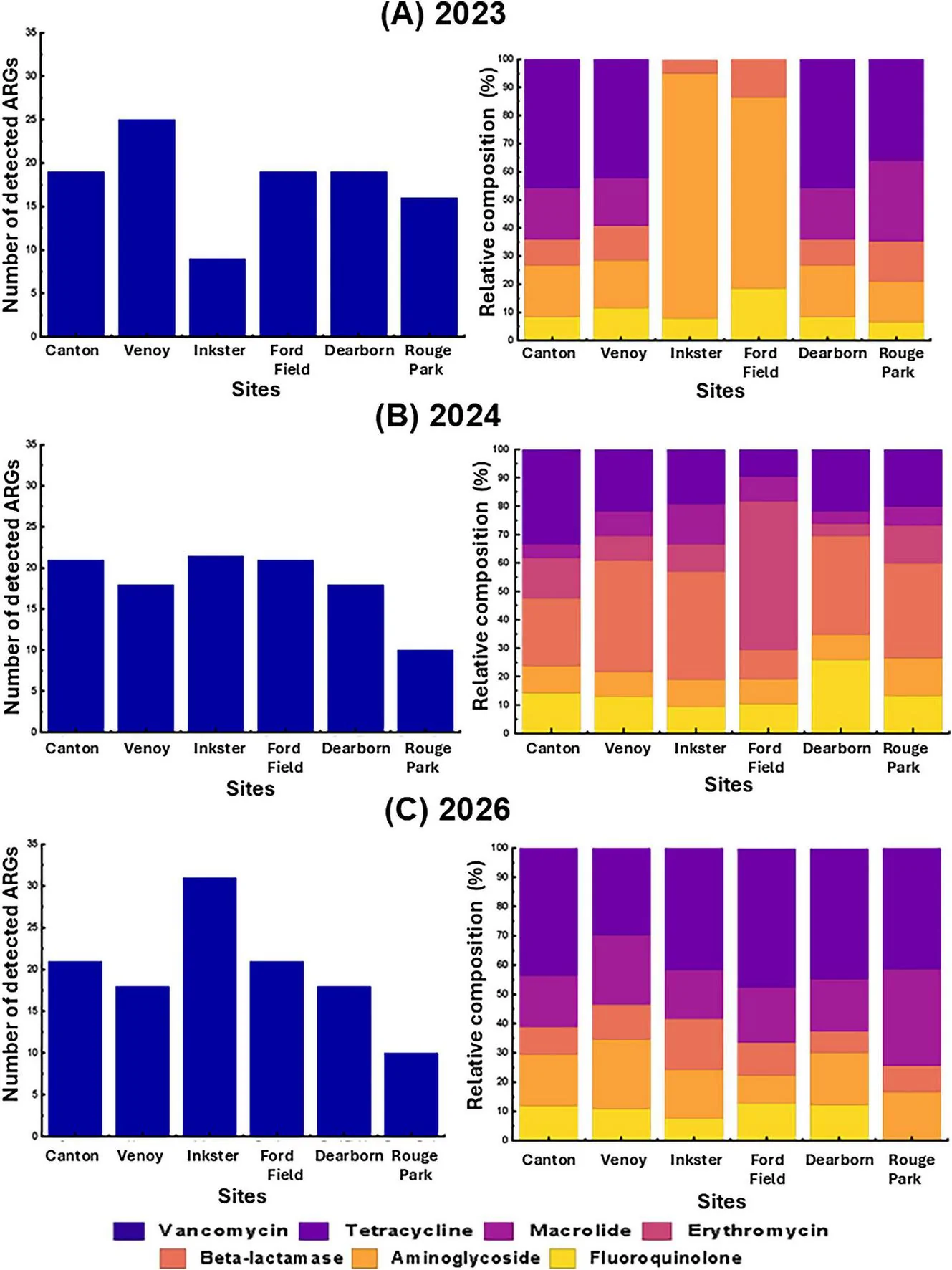
Bar charts showing the number of positively detected antibiotic resistance genes (left panels) and stacked bar charts showing the relative composition of antibiotic resistance classes (right panels) across six Rouge River sampling sites in 2023, 2024, and 2026. ARG richness varied among sampling sites and years, with Venoy showing the greatest number of detected ARGs in 2023 and Inkster in 2024 and 2026. The relative composition of resistance classes also varied spatially and temporally, with vancomycin and tetracycline resistance genes representing major components across multiple sampling sites. Detection frequency and relative composition of antibiotic resistance genes across six Rouge River sampling sites during **(A)** 2023, **(B)** 2024, and **(C)** 2026. Left panels show the total number of positively detected antibiotic resistance genes (ARGs; Cq ≤ 35) at each sampling site. Right panels show the relative composition of detected ARGs grouped according to antibiotic resistance class. Sampling sites are arranged along the upstream-to-downstream urbanization gradient from Canton to Rouge Park.

When expressed as a proportion of all positively detected ARGs, β-lactam resistance genes made the largest contribution to the overall ARG profiles, particularly at Inkster and Dearborn during 2024 and 2026. This pattern partly reflects the comparatively large number of β-lactamase targets represented on the array. Vancomycin resistance genes were detected at all sampling sites during the study period, whereas tetracycline, aminoglycoside, fluoroquinolone, macrolide, and erythromycin resistance genes showed greater variation among sites and sampling campaigns. Overall, ARG richness and class composition varied across the watershed. Notably, Inkster exhibited the greatest ARG richness during both 2024 and 2026 (31 detected genes) despite having an intermediate level of watershed urbanization.

#### 3.4.2 Spatial distribution of individual antibiotic resistance genes

Heat maps generated for each sampling campaign revealed pronounced variation among sites and sampling campaigns in the distribution of individual antibiotic resistance genes (ARGs) across the Rouge River watershed (Figure 10). In 2023, positive detections (Cq ≤ 35) were distributed throughout the watershed, with Venoy exhibiting the greatest number of detected ARGs. Representative aminoglycoside (*aadA1*), β-lactamase (OXA-10 and OXA-2 groups), fluoroquinolone [*AAC(6’)-Ib-cr*], macrolide (*ermB*), and tetracycline (*tetA*) resistance genes were detected at most sampling sites, whereas many β-lactamase genes exhibited site-specific positive or inconclusive detections.

**FIGURE 10:**
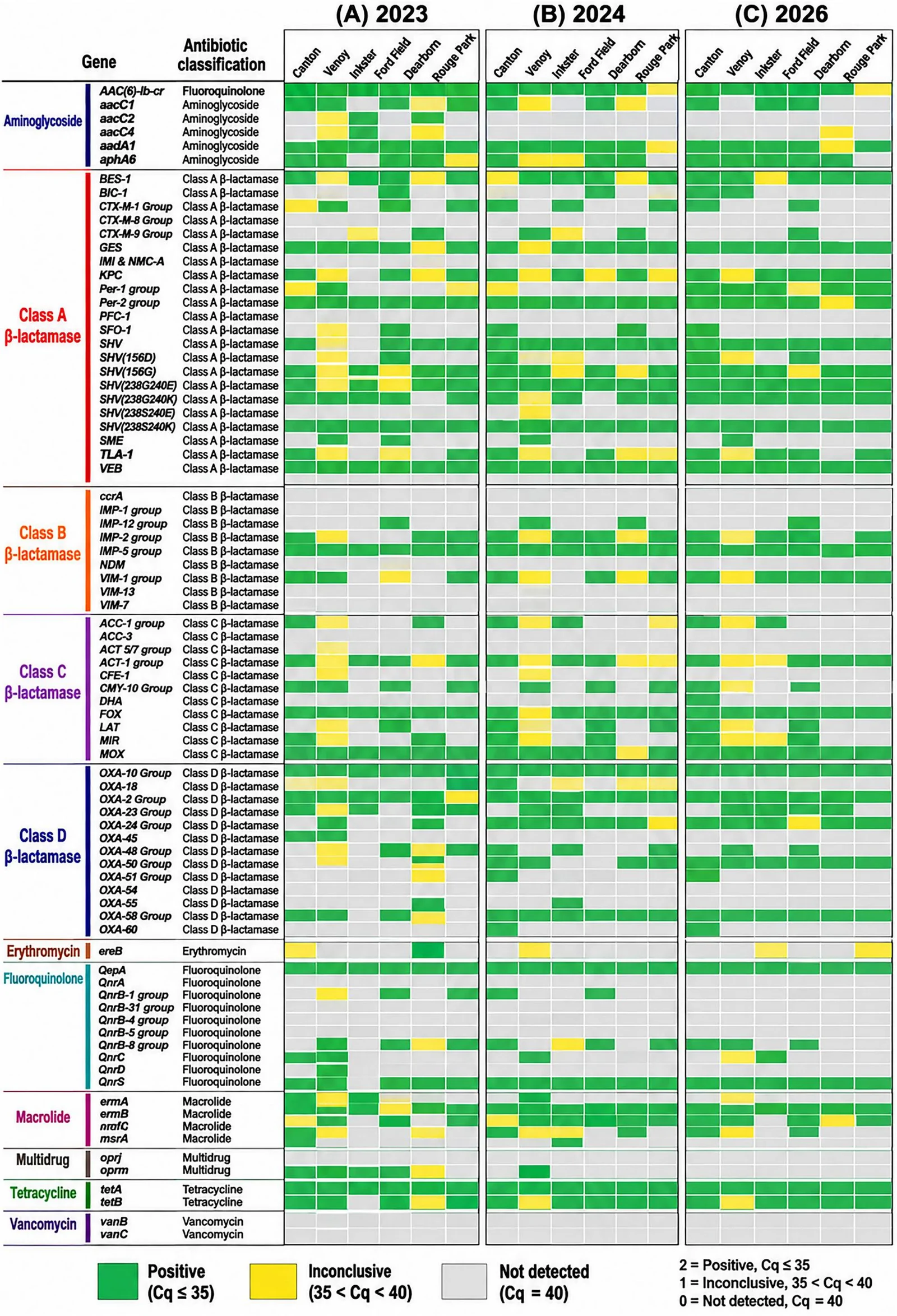
Three heat maps showing qPCR detection of 83 antibiotic resistance genes (ARGs) across six Rouge River sampling sites during 2023, 2024, and 2026. Rows represent individual ARGs grouped into antibiotic resistance classes, and columns represent sampling sites arranged from upstream (Canton) to downstream (Rouge Park). Green cells indicate positive detection (Cq ≤ 35), yellow cells indicate inconclusive detection (Cq > 35 and < 40), and light gray cells indicate no detection (Cq = 40). Spatial distribution of antibiotic resistance genes (ARGs) across six Rouge River sampling sites during **(A)** 2023, **(B)** 2024, and **(C)** 2026. Heat maps summarize qPCR detection of 83 ARGs grouped into antibiotic resistance classes. Rows represent individual ARGs, and columns represent sampling sites arranged from upstream (Canton) to downstream (Rouge Park). Green indicates positive detection (Cq ≤ 35), yellow indicates inconclusive detection (Cq > 35 and < 40), and light gray indicates no detection (Cq = 40).

Antibiotic resistance gene detections increased at the midstream sites in 2024, particularly at Inkster and Dearborn, where numerous β-lactamase genes were positively detected. Aminoglycoside, macrolide, fluoroquinolone, and tetracycline resistance genes remained broadly distributed throughout the watershed. Similar spatial patterns were observed in 2026, with Inkster continuing to exhibit the greatest number of detected ARGs, whereas Rouge Park consistently contained fewer positive detections. Overall, although several ARGs were consistently detected across the three sampling campaigns, numerous β-lactamase and fluoroquinolone resistance genes exhibited pronounced site-specific variation, contributing to the observed spatial heterogeneity in ARG profiles.

#### 3.4.3 Cq distributions of positively detected antibiotic resistance genes

The distribution of Cq values for positively detected antibiotic resistance genes (ARGs; Cq ≤ 35) varied among sampling sites, resistance classes, and sampling campaigns (Figure 11). In 2023, Inkster and Dearborn exhibited the greatest variability in Cq values, whereas Canton, Ford Field, and Rouge Park displayed narrower distributions with higher median Cq values. In 2024, Cq values were more uniformly distributed among sampling sites, although Inkster and Ford Field continued to exhibit greater variability than the remaining sites. In 2026, most Cq values were concentrated between 30 and 34 across all sampling sites, indicating relatively consistent amplification patterns among positively detected ARGs.

**FIGURE 11:**
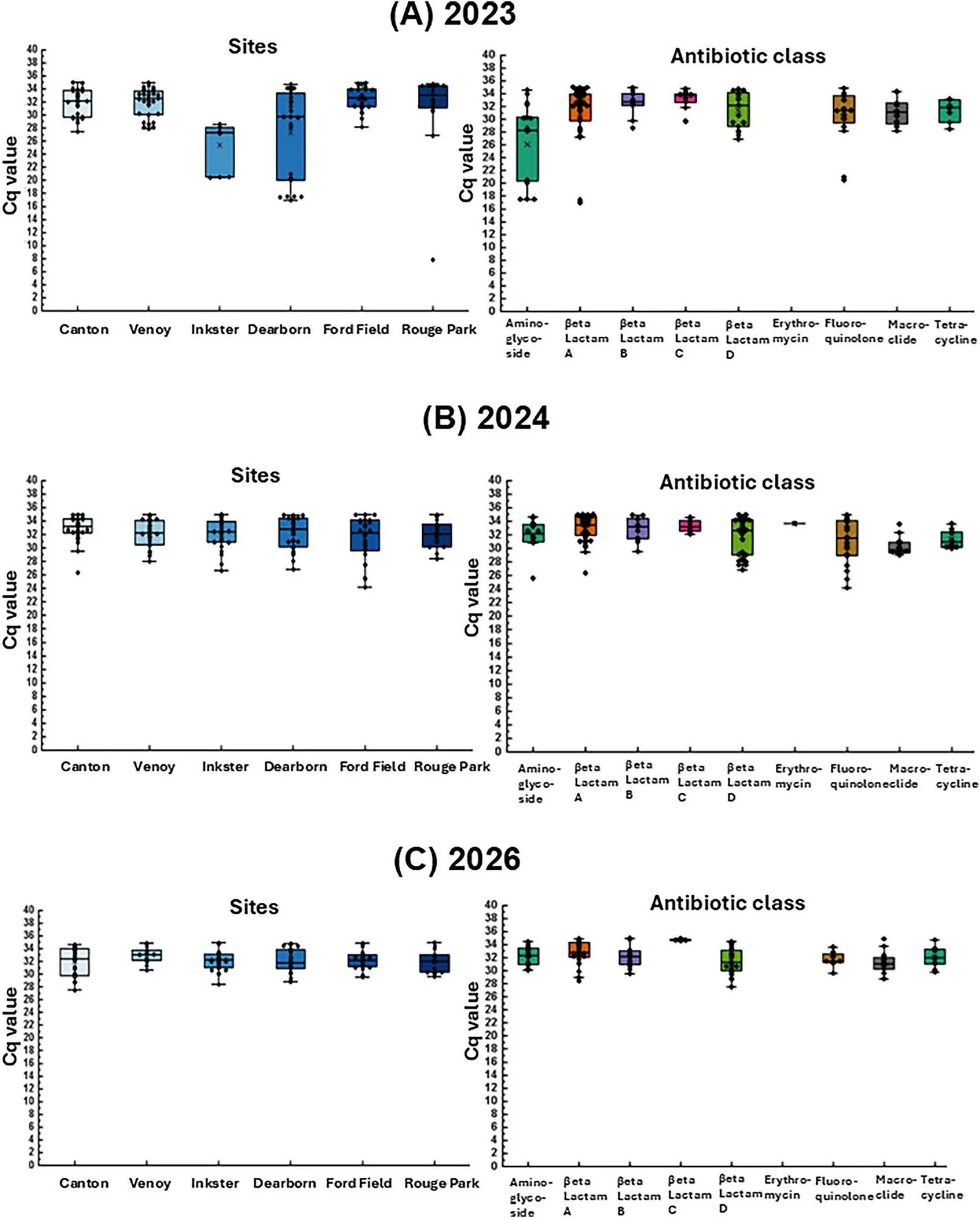
Six box-and-whisker plots arranged in three rows for 2023, 2024, and 2026. The left panels compare qPCR Cq values of positively detected antibiotic resistance genes (ARGs; Cq ≤ 35) among six Rouge River sampling sites, and the right panels compare Cq values among antibiotic resistance classes. Boxes represent the interquartile range, the horizontal line indicates the median, whiskers extend to 1.5 × IQR, and points represent observations outside the whiskers. The plots show the distribution and variability of Cq values among sampling sites and antibiotic resistance classes for each sampling campaign. Distribution of qPCR Cq values for positively detected antibiotic resistance genes (ARGs) across sampling sites and antibiotic resistance classes during **(A)** 2023, **(B)** 2024, and **(C)** 2026. Box-and-whisker plots summarize the distribution of Cq values for positively detected ARGs (Cq ≤ 35). The left panels compare sampling sites, whereas the right panels compare antibiotic resistance classes. Boxes represent the interquartile range (IQR), the horizontal line indicates the median, whiskers extend to 1.5 × IQR, and points represent observations outside the whiskers. Cq values represent amplification characteristics of positively detected targets within the standardized DNA input and are not interpreted as measures of relative or absolute environmental abundance.

Differences in Cq value distributions were also evident among ARG classes. Aminoglycoside and Class D β-lactamase genes exhibited the greatest variability across the three sampling campaigns, whereas erythromycin, macrolide, and tetracycline resistance genes showed narrower distributions with relatively consistent median Cq values. Fluoroquinolone and several β-lactamase classes displayed greater variation among sampling campaigns, reflecting differences in Cq distributions among positively detected targets. Together, Cq value distributions indicated generally similar amplification patterns among sampling sites, with greater variability associated primarily with specific resistance classes and sampling campaigns.

#### 3.4.4 Hierarchical clustering of antibiotic resistance class profiles

Hierarchical clustering revealed sampling campaign-specific similarities in ARG class profiles among sampling locations (Figure 12). In 2023, Canton and Ford Field formed the most similar cluster, whereas Inkster and Dearborn had more distinct class profiles. In 2024, Inkster, Rouge Park, and Ford Field clustered together, while Dearborn formed a separate branch characterized by a greater relative representation of tetracycline-associated ARGs. In 2026, Canton and Ford Field again clustered closely, whereas Venoy and Inkster formed a second cluster with similar class distributions.

**FIGURE 12:**
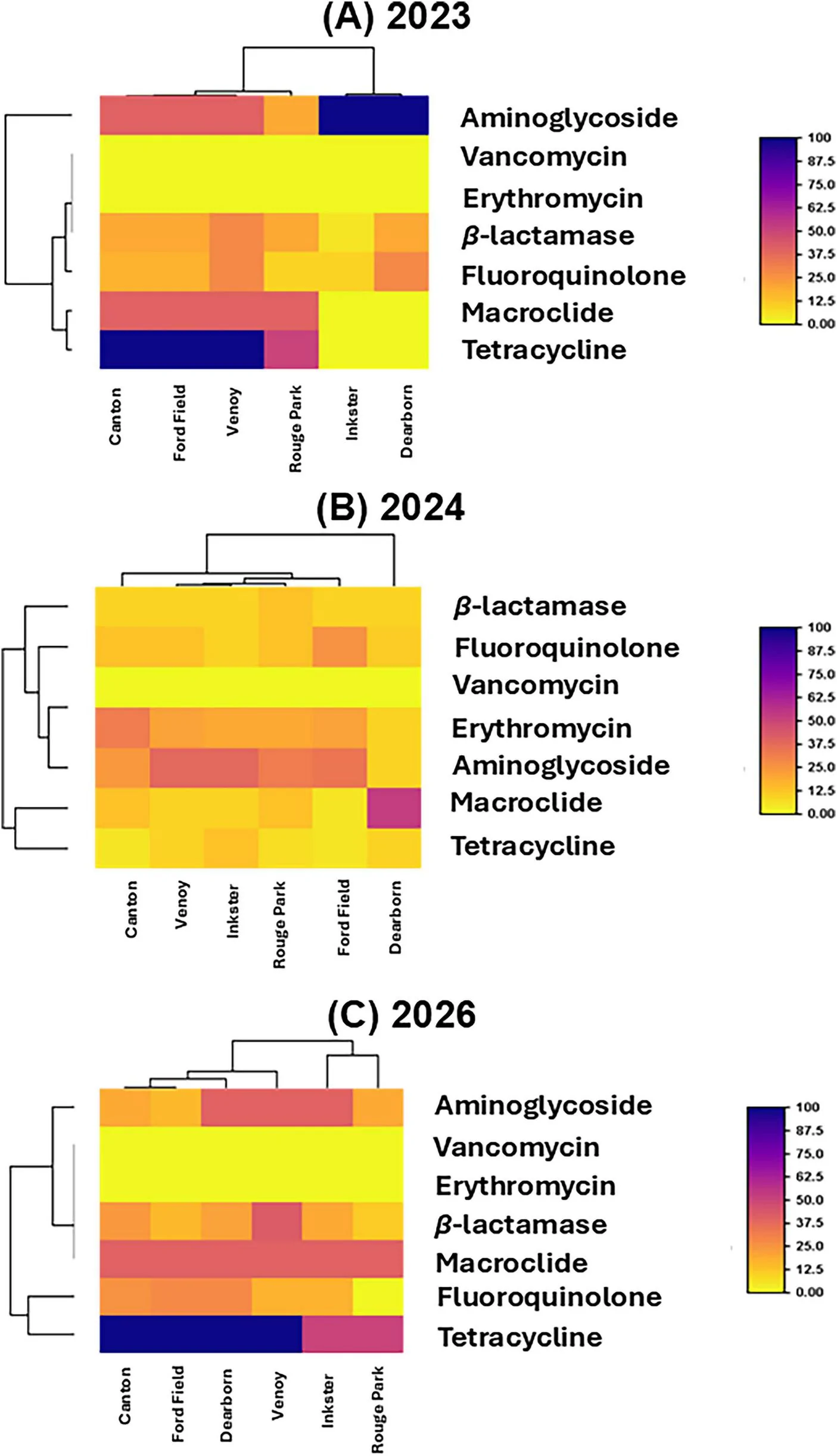
Three hierarchical clustering heat maps showing the class-normalized detection frequency of seven antibiotic resistance gene (ARG) classes across six Rouge River sampling sites during 2023, 2024, and 2026. Rows represent ARG classes, columns represent sampling sites arranged from upstream (Canton) to downstream (Rouge Park), and dendrograms indicate hierarchical clustering of similar ARG detection patterns. Cell color ranges from yellow (low class-normalized detection frequency) to blue (high class-normalized detection frequency). Hierarchical clustering heat maps of antibiotic resistance gene (ARG) classes across six Rouge River sampling sites during **(A)** 2023, **(B)** 2024, and **(C)** 2026. Heat maps show the class-normalized detection frequency (%) of positively detected ARGs (Cq ≤ 35) within each ARG class at each sampling site. Rows represent ARG classes, columns represent sampling sites arranged from upstream (Canton) to downstream (Rouge Park), and dendrograms illustrate hierarchical clustering based on similarities in ARG detection patterns. Color intensity represents class-normalized detection frequency, ranging from low (yellow) to high (blue).

When expressed as class-normalized detection frequency, which accounts for differences in the number of targets represented within each resistance class, tetracycline-associated ARGs exhibited the highest values across the three sampling campaigns. Vancomycin resistance genes had among the lowest class-normalized detection frequencies, whereas β-lactamase, fluoroquinolone, aminoglycoside, macrolide, and erythromycin resistance classes showed intermediate values. Thus, β-lactamase genes contributed the greatest number of total positive detections, whereas tetracycline-associated ARGs exhibited the highest class-normalized detection frequency.

#### 3.4.5 Multivariate ordination of antibiotic resistance gene profiles

Multivariate ordination analyses revealed variation in ARG composition among sampling sites and sampling campaigns and associations with the measured watershed and physicochemical variables (Figure 13). Non-metric multidimensional scaling (NMDS) showed partial overlap among samples, indicating gradual compositional turnover rather than distinct separation by sampling site or sampling campaign (stress = 0.090; Figure 13A). Redundancy analysis (RDA) showed that the selected watershed and physicochemical variables collectively explained 53.5% of the variation in ARG composition (adjusted R^2^ = 0.210). However, when the repeated site structure was accounted for in the permutation analysis, the overall RDA was not statistically significant (*P* ≈ 0.18; Figure 13B). The first two constrained axes, RDA1 and RDA2, were also not statistically significant (*P* ≈ 0.43 and *P* ≈ 0.34, respectively). The adjusted R^2^ indicates that approximately 21% of the variation was explained after accounting for the number of explanatory variables in the model. Accordingly, the RDA was interpreted as an exploratory ordination showing potential associations between ARG composition and the measured environmental variables rather than evidence of statistically significant environmental effects. These gradients included developed land cover, stream density, conductivity, dissolved oxygen, pH, turbidity, and total animal abundance. Thus, although ARG profiles did not form distinct site- or sampling campaign-specific clusters in the NMDS ordination, the RDA suggested potential associations between ARG composition and the measured environmental gradients, but these associations were not statistically significant after accounting for the repeated site structure.

**FIGURE 13:**
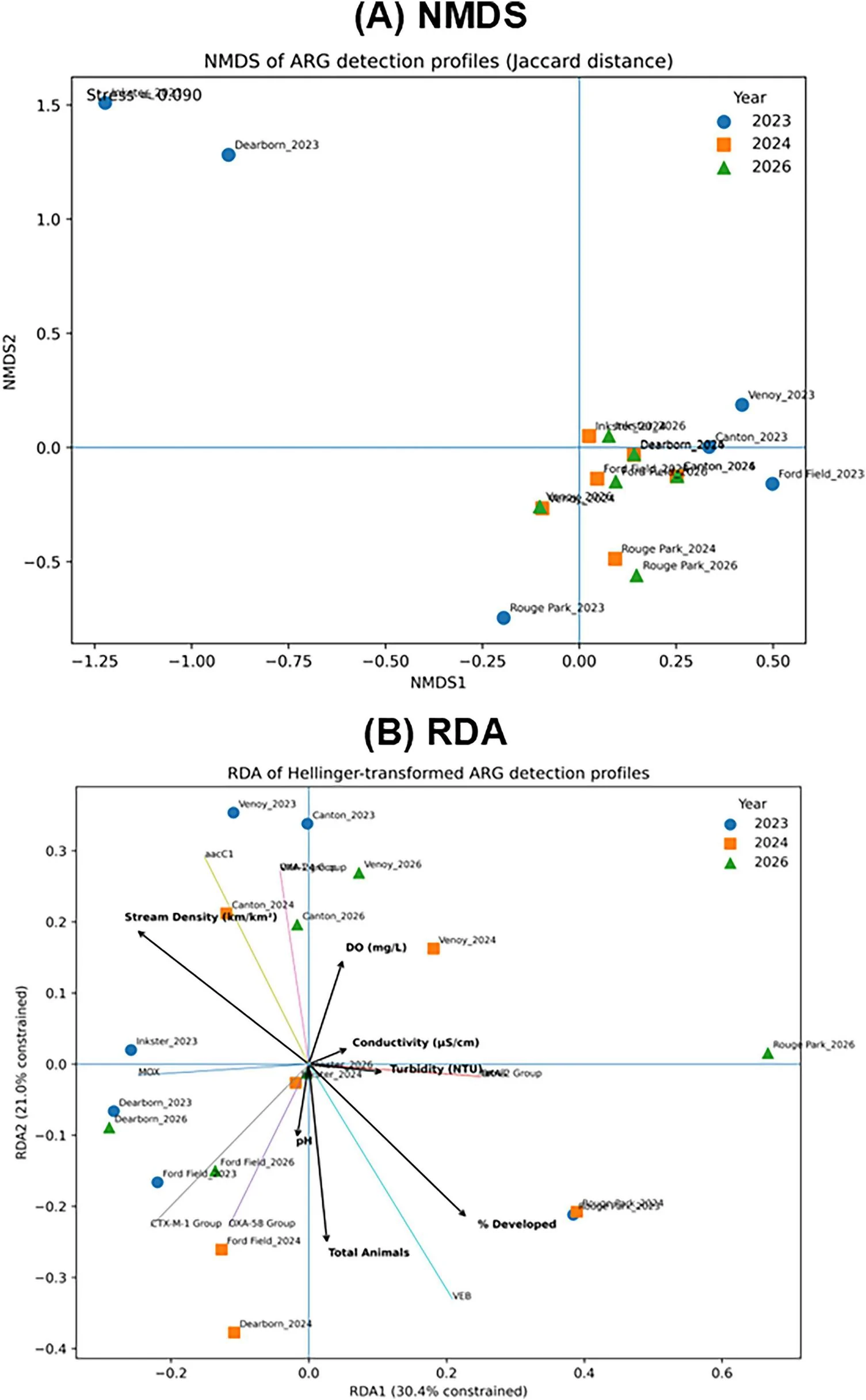
Two-panel ordination Figure showing antibiotic resistance gene (ARG) composition across six Rouge River sampling sites sampled in 2023, 2024, and 2026. **(A)** Shows a non-metric multidimensional scaling (NMDS) ordination based on Jaccard dissimilarity with individual site-by-sampling-campaign samples. **(B)** Shows a redundancy analysis (RDA) ordination of Hellinger-transformed ARG detection data with arrows representing selected watershed characteristics and physicochemical variables and their exploratory associations with ARG composition. Multivariate ordination of antibiotic resistance gene (ARG) composition across six Rouge River sampling sites during 2023, 2024, and 2026. **(A)** Non-metric multidimensional scaling (NMDS) ordination based on Jaccard dissimilarity showing similarities in ARG composition among sampling sites and sampling campaigns (stress = 0.090). **(B)** Redundancy analysis (RDA) of Hellinger-transformed ARG detection data showing relationships between ARG composition and selected watershed characteristics and physicochemical variables. Arrows represent environmental variables, with vector direction indicating the direction of association and vector length indicating the relative strength of the relationship. The environmental vectors represent exploratory associations and do not indicate statistically significant individual effects. The overall RDA was not statistically significant after accounting for repeated observations within sites (*P* ≈ 0.18).

Supporting ordination analyses provided additional context (Supplementary Figure 2). Principal coordinates analysis (PCoA) showed a sample distribution similar to the NMDS ordination, with the first two axes explaining 50.8% of the total variation in ARG composition (Supplementary Figure 2A). Canonical correspondence analysis (CCA) explained 46.7% of the constrained variation but was not statistically significant (*P* = 0.173; Supplementary Figure 2B). Thus, neither constrained ordination provided statistically significant evidence of an overall relationship between ARG composition and the measured environmental variables. The RDA nevertheless provided a descriptive representation of potential environmental associations, whereas these relationships should not be interpreted as statistically significant effects of individual environmental variables. In contrast to microbial water quality marker composition, which showed no significant relationship with the measured environmental variables, ARG composition likewise showed no statistically significant overall environmental association after accounting for the repeated site structure, although the RDA indicated exploratory relationships with several measured environmental gradients.

### 3.5 Exploratory correlations between watershed characteristics and molecular richness

Exploratory Pearson correlation analysis identified several associations between watershed characteristics and antibiotic resistance gene (ARG) richness, whereas microbial water quality marker richness showed no significant relationships with the measured environmental variables (Supplementary Table 4). ARG richness was negatively correlated with developed land cover, elevation, and watershed slope and positively correlated with forest cover and stream density. In contrast, microbial water quality marker richness was not significantly correlated with watershed characteristics or physicochemical conditions. Total animal abundance, precipitation, and water depth were also not significantly associated with either ARG richness or microbial water quality marker richness. These contrasting patterns indicate that ARG richness and microbial water quality marker richness differed in their associations with the measured watershed characteristics, consistent with the differences observed in the multivariate ordination analyses.

Because site-level watershed characteristics were repeated across three sampling campaigns, these correlations should be interpreted as exploratory and should not be considered evidence of causal relationships. In particular, the negative correlation between developed land and ARG richness indicates that greater developed land was not associated with greater ARG richness across these observations, rather than demonstrating that urbanization reduces ARG richness.

## 4 Discussion

### 4.1 Principal findings

This study demonstrated pronounced variation among sampling sites and campaigns in physicochemical conditions, microbial water quality markers, and antibiotic resistance gene (ARG) profiles across an urbanization gradient in the Rouge River watershed. Although both molecular datasets exhibited substantial variation among sites and sampling campaigns, their observed relationships with the measured environmental gradients differed. Microbial water quality marker composition was not significantly associated with the measured watershed characteristics or physicochemical variables, whereas the RDA of ARG composition indicated potential associations with these environmental gradients, although the overall relationship was not statistically significant after accounting for repeated observations within sites. Notably, Inkster exhibited the greatest ARG richness in both 2024 and 2026 despite having an intermediate proportion of developed land, identifying this midstream reach as a potential resistance hotspot. β-Lactamase genes contributed the largest proportion of ARG detections, whereas tetracycline-associated genes exhibited the highest normalized detection frequency within their resistance class. Collectively, these findings suggest that microbial water quality markers may reflect more localized or episodic contamination processes, whereas ARG profiles exhibited exploratory associations with the measured watershed and environmental gradients. The contrasting patterns exhibited by these molecular indicators demonstrate that ARGs and microbial water quality markers provide complementary information about urban watershed conditions. Their direct comparison using the same samples and environmental framework represents a central contribution of this study and highlights the value of integrating high-throughput qPCR, GIS-derived watershed characteristics, and physicochemical measurements for watershed-scale surveillance.

### 4.2 Relationships between urbanization, watershed characteristics, and antibiotic resistance gene composition

Antibiotic resistance gene (ARG) composition varied across the Rouge River watershed and showed exploratory associations with the measured watershed and physicochemical gradients in the RDA. However, the overall RDA was not statistically significant after accounting for repeated observations within sites, indicating that these relationships should be interpreted as potential environmental associations rather than statistically supported effects. The RDA nevertheless indicated exploratory associations between the measured environmental variables and differences in ARG profiles among sampling sites and sampling campaigns. However, this relationship was not expressed as a simple upstream-to-downstream increase in ARG richness. Consistent with this non-linear pattern, exploratory Pearson correlations showed that ARG richness was negatively associated with developed land cover and positively associated with forest cover and stream density. These associations should be interpreted cautiously because site-level watershed characteristics were repeated across sampling campaigns and the correlations were exploratory (Supplementary Table 4). In particular, the negative association with developed land should not be interpreted as evidence that increasing urbanization reduces ARG richness. Rather, the absence of a simple positive relationship between developed land and ARG richness suggests that resistance patterns reflect multiple interacting watershed characteristics rather than urbanization intensity alone. This interpretation is consistent with previous studies showing that environmental antimicrobial resistance in urban watersheds is associated with multiple interacting environmental and watershed-scale processes (Sowah et al., 2022; O’Malley et al., 2023; Chen et al., 2025; Liu et al., 2025).

Physicochemical conditions also varied significantly among sites and sampling campaigns, with significant Site × Year interactions for all measured variables. These interactions indicate that the magnitude or direction of spatial differences in water temperature, dissolved oxygen, pH, conductivity, TDS, and turbidity varied among sampling campaigns. Thus, individual sites exhibited different physicochemical conditions among the three sampling campaigns. These differences among sampling campaigns provide important context for interpreting ARG patterns because resistance profiles were evaluated against environmental conditions that also differed among sites and sampling events. The significant association between ARG composition and the measured environmental gradients therefore reflects relationships across a heterogeneous watershed sampled under differing environmental conditions rather than a single, stable environmental gradient. The exploratory relationships between ARG composition and the measured environmental gradients therefore represent patterns across a heterogeneous watershed sampled under differing environmental conditions rather than evidence of a single, statistically supported environmental gradient.

β-Lactamase genes contributed the largest proportion of ARG detections, particularly at Inkster and Dearborn during 2024 and 2026. This pattern is consistent with the frequent occurrence and, in some urban rivers, dominance of β-lactam resistance determinants in wastewater-influenced and urban aquatic environments (Wu et al., 2022; Kasuga et al., 2022). The relatively large number of β-lactamase targets represented on the qPCR array likely also contributed to their dominance in the overall ARG profile. In contrast, tetracycline-associated genes exhibited the highest detection frequency after normalization to the number of targets within each resistance class, suggesting that tetracycline resistance was especially widespread relative to the size of that target group. The broad occurrence of both β-lactam and tetracycline resistance genes is consistent with high-throughput surveys of urban and watershed rivers, although the dominant resistance classes differ among aquatic systems (Fernanda et al., 2022; Morina and Franklin, 2023). Previous isolate-based work also demonstrated the occurrence of aminoglycoside resistance determinants in urban watersheds, including the Rouge River. Catalfano et al. (2025) identified *aadA1*, *aadB*, and *aacA4* gene cassettes within class 1 integrons of *Escherichia coli* strains isolated from three urban watersheds. Together with the broader ARG profiles detected in the present study, these findings provide complementary evidence that urban watershed environments can harbor diverse antibiotic resistance determinants.

The occurrence of the greatest ARG richness at Inkster in both 2024 and 2026 further illustrates the absence of a simple relationship between ARG richness and urbanization intensity. Because Inkster had an intermediate proportion of developed land, its elevated ARG richness suggests that localized environmental conditions, watershed position, and other site-specific processes may contribute to spatial patterns in resistance. The potential mechanisms underlying this recurring midstream hotspot are considered in greater detail in section “4.5 Potential factors associated with the midstream ARG hotspot at Inkster.”

Collectively, these findings indicate that ARG patterns across the Rouge River showed potential relationships with multiple watershed and physicochemical characteristics but were not explained by a uniform downstream urbanization gradient. Importantly, these relationships are associative and should not be interpreted as evidence that developed land, physicochemical conditions, or any individual watershed characteristic caused the observed ARG distributions. Although the RDA ordination suggested relationships between ARG composition and several measured environmental gradients, the corrected overall permutation test was not statistically significant, and the environmental vectors should therefore be interpreted as exploratory associations rather than significant individual effects. The RDA suggested exploratory relationships between ARG composition and the measured environmental gradients; however, the corrected overall permutation test was not statistically significant, whereas the non-linear spatial patterns in ARG richness demonstrate that urbanization intensity alone did not predict ARG richness. Unmeasured factors, including localized wastewater or stormwater inputs, hydrologic transport, sediment-associated processes, and other site-specific conditions, may also have contributed to the observed patterns.

### 4.3 Microbial water quality markers respond differently

Microbial water quality marker composition exhibited substantial variation among sites and sampling campaigns but was not significantly associated with the measured watershed characteristics or physicochemical conditions. The lack of significant relationships in the constrained ordination analyses indicates that the measured environmental gradients did not systematically account for variation in microbial marker composition. Unlike ARGs, microbial marker profiles may therefore reflect localized, episodic, or source-specific contamination processes more strongly than the watershed-scale gradients measured in this study. This contrast suggests that microbial water quality markers and ARGs capture different dimensions of watershed condition and should be viewed as complementary rather than interchangeable indicators.

Waterborne pathogens, particularly *Aeromonas* spp. and *A. butzleri*, were widely distributed across sites and sampling campaigns. Their broad occurrence may reflect persistence in freshwater environments, sediments, and biofilms rather than a single recent contamination source (Battin et al., 2016; Nascimento et al., 2024). *Arcobacter* spp. have also been reported in urban stormwater and wastewater-impacted waters (Carson et al., 2024). In contrast, human fecal-associated markers and opportunistic pathogens were detected less consistently, whereas animal-associated markers occurred more frequently, suggesting contributions from multiple localized and environmental sources. Differences among sampling campaigns may also partly reflect episodic hydrologic or stormwater inputs that were not fully captured by the environmental variables measured during individual sampling events (Ahmed et al., 2021). This interpretation is consistent with the hierarchical clustering analysis, which showed sampling campaign-specific differences in similarities among sampling sites and microbial water quality marker categories (Figure 7).

Overall, the microbial water quality marker results indicate variable patterns of microbial marker detection among sites and sampling campaigns that were not systematically associated with the measured watershed and physicochemical gradients. In contrast, ARG composition exhibited a significant overall association with these environmental gradients, reinforcing the conclusion that the two molecular indicator groups respond differently to environmental conditions across the watershed.

### 4.4 Why do antibiotic resistance genes and microbial water quality markers respond differently?

One of the principal findings of this study was that antibiotic resistance genes (ARGs) and microbial water quality markers exhibited different patterns in relation to the same urban watershed gradient. Although both molecular datasets were generated from the same water samples and evaluated against identical watershed characteristics and physicochemical variables, their ordination patterns differed, although neither dataset showed a statistically significant overall relationship with the measured environmental gradients after accounting for the repeated site structure (Figures 8, 13). The RDA nevertheless indicated exploratory associations between ARG composition and several measured environmental variables that were not evident for the microbial water quality markers. This contrast suggests that the two groups of molecular indicators are influenced by different ecological processes and may reflect different ecological processes and environmental influences.

Microbial water quality markers indicate the presence of specific organisms or source-associated genetic targets and are commonly used to identify potential fecal-contamination sources. Their occurrence may vary with sewage inputs, wildlife activity, stormwater runoff, wastewater discharges, and short-term hydrologic conditions, as demonstrated by differences between baseflow and stormflow conditions in urban rivers (Ahmed et al., 2021). However, detection does not necessarily indicate a recent contamination event because marker DNA can persist during wastewater treatment and after discharge into surface waters (Lobos et al., 2024). Microbial marker profiles may therefore reflect both episodic inputs and marker-specific environmental persistence, which could weaken their association with stable watershed-scale characteristics.

In contrast, ARGs can occur within viable bacteria, extracellular DNA, sediments, biofilms, and mobile genetic elements. Wastewater-associated ARGs may persist in river sediments following particle deposition, providing environmental reservoirs from which resistance determinants may later be remobilized (Brown et al., 2020). Plasmids and other mobile genetic elements also facilitate horizontal transfer of resistance determinants among bacterial populations and across human, animal, and environmental compartments (Castañeda-Barba et al., 2024). ARG composition may therefore integrate the cumulative effects of anthropogenic inputs, environmental selection, hydrologic transport, and genetic exchange over longer periods than individual microbial-marker detections. This interpretation is consistent with the exploratory relationships between ARG composition and several measured watershed and physicochemical gradients observed in the RDA, although the corrected overall permutation test was not statistically significant (Figure 13B).

These contrasting responses have important implications for watershed surveillance. Microbial water quality markers provide information about potential contamination sources and the distribution of source-associated microorganisms, whereas ARGs characterize targeted resistance profiles and their relationships with watershed conditions. Integrating both datasets therefore provides a more comprehensive assessment than either approach alone by linking microbial-contamination patterns with the occurrence and distribution of targeted resistance determinants. Such integration is consistent with the One Health view that environmental antimicrobial resistance connects human, animal, and environmental systems through anthropogenic contamination, environmental reservoirs, and gene transfer (Raziq et al., 2026).

Previous urban-river studies have characterized ARG distributions using high-throughput molecular analyses or examined relationships between sewage-associated markers, ARGs, and hydrologic conditions (Ahmed et al., 2021; Wu et al., 2022; Kasuga et al., 2022). To our knowledge, however, direct comparisons of broad microbial water quality marker and ARG response matrices using the same samples, environmental variables, and multivariate framework remain limited. By demonstrating that these molecular datasets exhibited different compositional and ordination patterns within the same watershed framework, the present study highlights the value of integrating microbial water quality markers, ARG profiling, GIS-derived watershed characteristics, and physicochemical measurements in urban watershed surveillance.

### 4.5 Potential factors associated with the midstream ARG hotspot at Inkster

One of the most notable findings of this study was the repeated occurrence of the greatest antibiotic resistance gene (ARG) richness at Inkster in both 2024 and 2026. This pattern was unexpected because Inkster was not the most highly urbanized site within the Rouge River watershed, indicating that the observed ARG distribution was not explained by the overall percentage of developed land alone. The repeated observation of elevated ARG richness at Inkster in the 2024 and 2026 sampling campaigns suggests that localized watershed processes may contribute to the observed pattern at this site. Collectively, these findings suggest that watershed position, hydrologic connectivity, and localized inputs may contribute to the observed ARG richness patterns beyond what can be explained by urban land cover alone.

Antecedent precipitation differed among sampling campaigns at Inkster. Cumulative precipitation during the 72 h preceding sampling was 20.83 mm in 2023, 4.83 mm in 2024, and 9.40 mm in 2026 (Supplementary Table 2). Notably, the elevated ARG richness observed in 2024 and 2026 occurred under contrasting antecedent rainfall conditions, suggesting that recent precipitation alone is unlikely to explain the repeated observation of elevated ARG richness. The Inkster reach is also located within an urban corridor containing permitted combined-sewer systems that discharge to the Lower Rouge River [U.S. Environmental Protection Agency (U.S. EPA), 2026]. These potential wastewater and stormwater inputs provide additional context for the observed pattern; however, the present study was not designed to attribute ARG occurrence to specific discharge sources.

One possible explanation for the Inkster pattern is its position within the watershed, where the river integrates inputs from multiple upstream areas. Such hydrologic connectivity could facilitate the transport of resistant bacteria, extracellular ARGs, and other contaminants derived from urban, suburban, and agricultural sources. Stormwater runoff and urban drainage networks may further enhance this transport during precipitation events by mobilizing microorganisms, sediments, nutrients, and associated ARGs (Ahmed et al., 2021; O’Malley et al., 2023; Wu et al., 2022). However, the occurrence of elevated ARG richness at Inkster under differing antecedent rainfall conditions indicates that storm-driven inputs alone are unlikely to account for the recurring pattern. The elevated richness at Inkster may therefore reflect a combination of cumulative watershed inputs and more persistent or recurrent localized sources or processes.

Environmental persistence may provide an additional mechanism. River sediments and biofilms can serve as reservoirs of antibiotic resistance because resistant bacteria, extracellular DNA, and mobile genetic elements can accumulate on particles and within biofilm matrices, where they may be protected from degradation (Brown et al., 2020; Castañeda-Barba et al., 2024). Sediment resuspension during elevated flows could subsequently release these resistance determinants into the water column and increase the diversity of detectable ARGs. Although sediment dynamics were not directly measured in this study, these processes provide plausible mechanisms for the persistence and episodic mobilization of ARGs and warrant further investigation.

Overall, the Inkster pattern emphasizes that watershed position, hydrologic connectivity, and localized anthropogenic inputs should be considered alongside land use when evaluating factors potentially associated with ARG distribution.

### 4.6 One Health and implications for watershed monitoring

This study demonstrates the value of integrating high-throughput qPCR, GIS-derived watershed characteristics, physicochemical measurements, and multivariate analyses to evaluate microbial water quality and environmental antimicrobial resistance in urban watersheds. These complementary approaches capture different dimensions of watershed conditions, including land-use patterns, environmental gradients, microbial water quality markers, and targeted ARG profiles. Evaluating these datasets within a common analytical framework provides an integrated perspective on the relationships among watershed characteristics, microbial contamination, and environmental antimicrobial resistance.

The contrasting responses of microbial water quality markers and ARGs further illustrate the value of integrating multiple molecular indicators in watershed surveillance. Whereas microbial marker composition exhibited substantial variation among sites and sampling campaigns without a significant relationship to the measured environmental gradients, ARG composition exhibited exploratory relationships with several watershed characteristics and physicochemical gradients in the RDA, although the corrected overall relationship was not statistically significant after accounting for repeated observations within sites (Figures 8, 13). Together, these findings indicate that microbial water quality markers and ARGs provide complementary information about microbial contamination and environmental antimicrobial resistance. Similar integrated monitoring strategies have been recommended for environmental antimicrobial resistance surveillance because they can improve characterization of contamination sources and the environmental processes influencing resistance (Castañeda-Barba et al., 2024; Raziq et al., 2026).

From a One Health perspective, urban rivers represent interfaces where human activities, animal populations, and environmental processes interact to influence microbial water quality and antimicrobial resistance. Integrating molecular, environmental, and geospatial datasets therefore provides a framework for evaluating these interconnected factors and identifying areas where contamination and resistance may accumulate. The repeated observation of elevated ARG richness at Inkster in 2024 and 2026 further illustrates the potential value of identifying patterns that may not be predicted by broad measures of urbanization alone. However, qPCR detects target DNA regardless of whether it originates from viable cells, nonviable cells, or extracellular DNA. Thus, positive ARG detection under the operational Cq criterion used in this study demonstrates the environmental occurrence of targeted resistance determinants but does not establish the presence of viable resistant bacteria, expression of resistance, potential for horizontal transfer, or human exposure. The ARG patterns observed here should therefore be interpreted as environmental AMR surveillance signals rather than direct measures of transmission or public-health risk.

Within these interpretive boundaries, the findings support integrated watershed surveillance approaches that combine molecular, environmental, and geospatial information to identify spatial patterns of environmental antimicrobial resistance and prioritize locations for further investigation within a One Health framework (O’Malley et al., 2023; Castañeda-Barba et al., 2024; Tiquia-Arashiro et al., 2025b).

### 4.7 Limitations

Several limitations should be considered when interpreting these findings. High-throughput qPCR array analyses were performed using one pooled DNA sample per site-year, providing a composite molecular profile but precluding assessment of within-site variation. Filtration volumes varied from 300 to 650 mL because of filter clogging and were not used to normalize the molecular data. Although each array received the same 500-ng DNA input, this standardized template DNA mass but did not account for differences in microbial biomass, DNA yield, or source-water volume. Consequently, the qPCR results represent detection patterns and Cq distributions within the standardized DNA input and cannot be interpreted as absolute target concentrations or environmental ARG loads per unit volume of water.

Sampling was conducted during a single campaign in each of the three sampled years (2023, 2024, and 2026); therefore, the observed patterns represent discrete snapshots and cannot resolve seasonal variability or responses to individual storm events. Antecedent precipitation provided hydrologic context, but stream discharge and flow conditions were not measured directly. GIS-derived watershed characteristics represented single site-level spatial measurements and therefore did not capture potential changes in watershed characteristics among sampling campaigns. Because these site-level variables were repeated across the three sampling campaigns, the effective replication for evaluating their relationships with molecular composition was limited; accordingly, the constrained ordination results should be interpreted as exploratory.

The measured environmental variables did not encompass all potential drivers of ARG and microbial water quality marker composition, including antibiotic residues, heavy metals, nutrients, sediment characteristics, wastewater and stormwater outfall proximity, sewer density, discharge, and overflow events. Consequently, potential wastewater and stormwater influences should be considered plausible but unmeasured drivers rather than demonstrated sources. Pearson correlations were exploratory and represent associations rather than causal relationships.

Finally, the qPCR arrays targeted predefined sets of ARGs and microbial water quality markers and therefore do not characterize the complete environmental resistome or microbial community. In addition, the functional categories used to classify microbial water quality markers are not mutually exclusive, as individual targets may have multiple ecological or source associations. For example, *Campylobacter* and *Salmonella* may be associated with both animal sources and waterborne pathogens. Thus, the category-level patterns should be interpreted in the context of these overlapping ecological and source associations. Accordingly, the microbial water quality marker results should be interpreted as targeted molecular profiles rather than as measures of overall microbial community composition or diversity. Detection of target DNA also does not distinguish DNA derived from viable and nonviable cells or extracellular DNA and does not provide information on gene expression or mobility. These constraints limit inference regarding the biological activity and potential human-health implications of the detected ARGs.

Despite these limitations, integrating targeted molecular profiles with physicochemical and geospatial data provided a useful framework for evaluating watershed-scale patterns in microbial water quality and environmental antimicrobial resistance.

### 4.8 Future research

Future research should determine whether the spatial patterns identified in this study persist across seasons and hydrologic conditions and clarify the processes contributing to localized ARG enrichment. Repeated seasonal sampling and targeted sampling under both base-flow and wet-weather conditions, particularly at sites such as Inkster, would help distinguish persistent local patterns from event-specific variation. Incorporating hydrologic measurements, including stream discharge and flow conditions, together with wastewater and stormwater infrastructure mapping, discharge and overflow-event data, sediment characterization, and microbial source tracking would further help identify potential ARG sources and transport pathways.

Future molecular analyses should also extend beyond detection-based qPCR by incorporating quantitative measurements expressed relative to source-water volume, together with approaches that can evaluate bacterial viability, ARG mobility, and potential host associations. Measurement of additional environmental factors, including antibiotic residues, heavy metals, and nutrients, could further clarify selective pressures associated with ARG occurrence. Together, these approaches would help determine the persistence, sources, transport, and mobility of ARGs in urban watersheds. Identifying persistent ARG hotspots could also help prioritize locations for more intensive monitoring and source-tracking efforts, thereby strengthening environmental AMR surveillance within a One Health framework.

## 5 Conclusion

This study demonstrates that microbial water quality markers and antibiotic resistance genes (ARGs) exhibited contrasting spatial patterns and differences among sampling campaigns across the Rouge River watershed. Although microbial water quality marker composition exhibited substantial variation among sites and sampling campaigns, it was not significantly associated with the measured watershed characteristics or physicochemical variables. In contrast, ARG composition showed exploratory associations with several measured watershed and physicochemical gradients in the RDA, although the overall relationship was not statistically significant after accounting for repeated observations within sites. These patterns suggest that ARG distributions may reflect multiple watershed-scale characteristics and localized processes rather than land-use intensity alone.

By directly comparing microbial water quality markers and ARGs within the same samples using high-throughput qPCR integrated with GIS-derived watershed characteristics, physicochemical measurements, and multivariate analyses, this study demonstrates the complementary value of these targeted molecular indicators for watershed surveillance. The repeated observation of elevated ARG richness at Inkster in 2024 and 2026 further highlights the potential importance of localized watershed processes that may not be captured by broad measures of urbanization alone. Because the qPCR analyses detected target DNA but did not assess bacterial viability, ARG mobility or transferability, or human exposure, the observed ARG patterns should not be interpreted as direct measures of public-health risk. Rather, they provide indicators of environmental AMR occurrence that can support watershed-scale surveillance.

The distinct patterns observed for microbial water quality markers and ARGs highlight the value of integrating complementary molecular indicators with environmental and geospatial datasets to characterize antimicrobial resistance patterns in urban watersheds. Overall, the findings of this study advance our understanding of how urbanization shapes freshwater microbial ecosystems and support the integration of molecular, environmental, and geospatial datasets to strengthen watershed-scale surveillance and environmental risk assessment within a One Health framework.

## Data Availability

The original contributions presented in this study are included in this article/Supplementary material, further inquiries can be directed to the corresponding author.

## Supplementary material

Supplementary Figure 1 Two-panel supplementary ordination figure showing microbial water quality marker composition across six Rouge River sampling sites sampled in 2023, 2024, and 2026. **(A)** Shows a principal coordinates analysis (PCoA) based on Jaccard dissimilarity with individual site-year samples. **(B)** Shows a canonical correspondence analysis (CCA) of microbial water quality marker detection data with arrows representing selected watershed characteristics and physicochemical variables associated with microbial water quality marker composition. Supporting multivariate ordination analyses of microbial water quality marker composition across six Rouge River sampling sites during 2023, 2024, and 2026. **(A)** Principal coordinates analysis (PCoA) based on Jaccard dissimilarity showing similarities in microbial water quality marker composition among sampling sites and years. **(B)** Canonical correspondence analysis (CCA) of microbial water quality marker detection data showing relationships between microbial water quality marker composition and selected watershed characteristics and physicochemical variables. Arrows represent environmental variables, with vector direction indicating the direction of association and vector length indicating the relative strength of the relationship.

Supplementary Figure 2 Two-panel supplementary ordination figure showing antibiotic resistance gene (ARG) composition across six Rouge River sampling sites sampled in 2023, 2024, and 2026. **(A)** Shows a principal coordinates analysis (PCoA) based on Jaccard dissimilarity with individual site-year samples. **(B)** Shows a canonical correspondence analysis (CCA) of ARG detection data with arrows representing selected watershed characteristics and physicochemical variables associated with ARG composition. Supporting multivariate ordination analyses of antibiotic resistance gene (ARG) composition across six Rouge River sampling sites during 2023, 2024, and 2026. **(A)** Principal coordinates analysis (PCoA) based on Jaccard dissimilarity showing similarities in ARG composition among sampling sites and years. **(B)** Canonical correspondence analysis (CCA) of ARG detection data showing relationships between ARG composition and selected watershed characteristics and physicochemical variables. Arrows represent environmental variables, with vector direction indicating the direction of association and vector length indicating the relative strength of the relationship.
