# Supplementary material for: Urbanization shapes the landscape of antibiotic resistance genes and water quality microbial markers across an urban watershed
Source: Front Microbiol. 2026 Oct 9;17:1974267. doi: 10.3389/fmicb.2026.1974267 (PMC13690798; doi:10.3389/fmicb.2026.1974267)
Supplement: Supplementary file 1 [file Table_1.docx]

**Table S1.** Field conditions recorded during water sampling at six Rouge River sites in 2023, 2024, and 2026.

| **Site** | **Year** | **Air temperature**  **(^o^C)** | **Relative humidity**  **(%)** | **Atmospheric pressure**  **(atm)** | **Water depth**  **(cm)** | **Precipitation**  **(mm)** |
| --- | --- | --- | --- | --- | --- | --- |
| Canton | 2023 | 19.5 | 83.7 | 0.989 | 45.0 | 0.00 |
|  | 2024 | 22.7 | 41.0 | 1.006 | 12.0 | 0.00 |
|  | 2026 | 12.8 | 74.0 | 1.009 | 39.5 | 0.00 |
| Venoy | 2023 | 17.6 | 96.0 | 0.978 | 131.2 | 0.33 |
|  | 2024 | 24.4 | 66.0 | 1.000 | 22.0 | 0.00 |
|  | 2026 | 12.8 | 56.0 | 1.013 | 46.0 | 0.00 |
| Inkster | 2023 | 17.7 | 99.2 | 0.977 | 51.9 | 0.5 |
|  | 2024 | 25.0 | 68.0 | 0.999 | 21.0 | 0.00 |
|  | 2026 | 24.0 | 74.0 | 1.001 | 45.0 | 0.25 |
| Ford Field | 2023 | 25.1 | 70.2 | 0.977 | 64.2 | 0.25 |
|  | 2024 | 21.7 | 45.0 | 1.001 | 61.0 | 0.00 |
|  | 2026 | 12.8 | 53.0 | 1.003 | 64.5 | 0.00 |
| Dearborn | 2023 | 24.3 | 61.8 | 0.987 | 85.4 | 0.00 |
|  | 2024 | 24.0 | 64.0 | 1.001 | 51.0 | 0.00 |
|  | 2026 | 12.7 | 43.0 | 1.009 | 49.5 | 0.00 |
| Rouge Park | 2023 | 20.5 | 90.0 | 0.982 | 230.5 | 0.00 |
|  | 2024 | 16.7 | 71.0 | 0.982 | 80.0 | 0.05 |
|  | 2026 | 10.6 | 66.0 | 1.007 | 37.5 | 0.05 |

**Note:** Air temperature is reported as ambient meteorological context for each sampling campaign; site-specific water temperatures are reported in Table 2.
