# Supplementary material for: Urbanization shapes the landscape of antibiotic resistance genes and water quality microbial markers across an urban watershed
Source: Front Microbiol. 2026 Oct 9;17:1974267. doi: 10.3389/fmicb.2026.1974267 (PMC13690798; doi:10.3389/fmicb.2026.1974267)
Supplement: Supplementary file 2 [file Table_2.docx]

**Table S2.** Antecedent precipitation for water-sampling events at six Rouge River sites in 2023, 2024, and 2026.

| **Year** | **Site** | **Sampling date** | **Previous 24 h (mm)** | **Previous 48 h (mm)** | **Previous 72 h (mm)** |
| --- | --- | --- | --- | --- | --- |
| 2023 | Dearborn | June 21 | 0.00 | 0.00 | 0.00 |
| 2023 | Ford Field | June 23 | Trace | Trace | Trace |
| 2023 | Inkster | June 26 | 15.24 | 15.24 | 20.83 |
| 2023 | Venoy | June 27 | 22.10 | 37.34 | 37.34 |
| 2023 | Canton | June 28 | Trace | 22.10 | 37.34 |
| 2023 | Rouge Park | June 28 | Trace | 22.10 | 37.34 |
| 2024 | Dearborn | May 20 | 3.81 | 3.81 | 4.32 |
| 2024 | Venoy | May 21 | 1.02 | 4.83 | 4.83 |
| 2024 | Inkster | May 22 | 0.00 | 1.02 | 4.83 |
| 2024 | Canton | May 23 | 0.00 | 0.00 | 1.02 |
| 2024 | Ford Field | May 24 | 0.00 | 0.00 | 0.00 |
| 2024 | Rouge Park | May 28 | 9.40 | 27.94 | 30.23 |
| 2026 | Dearborn | May 12 | 0.00 | 0.00 | 0.51 |
| 2026 | Rouge Park | May 13 | 0.76 | 0.76 | 0.76 |
| 2026 | Ford Field | May 14 | 0.00 | 0.76 | 0.76 |
| 2026 | Inkster | May 19 | 7.87 | 7.87 | 9.40 |
| 2026 | Canton | May 20 | 16.76 | 24.64 | 24.64 |
| 2026 | Venoy | May 21 | 0.00 | 16.76 | 24.64 |

**Note.** Antecedent precipitation represents cumulative precipitation during the 1, 2, or 3 calendar days preceding each sampling date; precipitation on the sampling day is excluded. Values were obtained from the NOAA/NCEI Detroit Metropolitan Airport station (GHCND: USW00094847) to provide a consistent regional measure of antecedent rainfall across sampling events. Trace indicates precipitation reported as a trace amount. These data provide regional hydrometeorological context and may not capture localized rainfall at individual sampling sites.
