# Supplementary material for: Urbanization shapes the landscape of antibiotic resistance genes and water quality microbial markers across an urban watershed
Source: Front Microbiol. 2026 Oct 9;17:1974267. doi: 10.3389/fmicb.2026.1974267 (PMC13690798; doi:10.3389/fmicb.2026.1974267)
Supplement: Supplementary file 3 [file Table_3.docx]

**Table S3.** Functional classification of microbial water quality markers included in the Qiagen Microbial DNA Water Quality Array. Microbial markers were classified according to their primary ecological or contamination source association based on the Qiagen array annotation and published information on microbial ecology and host association.

| **Functional Category** | **Microbial Water Quality Marker** |
| --- | --- |
| **Human fecal-associated markers** | *Akkermansia muciniphila* |
|  | *Bacteroides thetaiotaomicron* |
|  | *Bacteroides vulgatus* |
|  | *Bifidobacterium adolescentis* |
|  | *Bifidobacterium dentium* |
|  | *Bifidobacterium longum* |
|  | *Lactobacillus gasseri* |
|  | *Ruminococcus bromii* |
|  | *Ruminococcus obeum* |
| **Human-associated opportunistic pathogens** | *Actinomyces gerencseriae* |
|  | *Actinomyces israelii* |
|  | *Citrobacter freundii* |
|  | *Clostridium difficile* |
|  | *Clostridium septicum* |
|  | *Helicobacter pylori* |
|  | *Morganella morganii* |
|  | *Staphylococcus aureus* |
|  | *Streptococcus agalactiae* |
| **Fecal indicator bacteria** | *Clostridium perfringens* |
|  | *Enterococcus casseliflavus* |
|  | *Enterococcus faecalis* |
|  | *Enterococcus faecium* |
|  | *Enterococcus gallinarum* |
| **Animal-associated markers** | *Campylobacter coli* |
|  | *Campylobacter jejuni* |
|  | *Campylobacter lari* |
|  | *Campylobacter subantarcticus* |
|  | *Campylobacter upsaliensis* |
|  | *Catellicoccus marimammalium* |
|  | *Streptococcus suis* |
|  | *Yersinia enterocolitica* |
|  | *Yersinia pestis* |
|  | *Yersinia pseudotuberculosis* |
| **Waterborne pathogens** | *Aeromonas enteropelogenes* |
|  | *Aeromonas hydrophila* |
|  | *Aeromonas media* |
|  | *Aeromonas punctata* |
|  | *Arcobacter butzleri* |
|  | *Arcobacter skirrowii* |
|  | *Salmonella enterica* |
|  | *Shigella dysenteriae* |
|  | *Vibrio cholerae* |
|  | *Vibrio parahaemolyticus* |
|  | *Vibrio vulnificus* |
| **Environmental source indicators** | *Desulfovibrio desulfuricans* |
|  | *Desulfovibrio vulgaris* |
|  | *Legionella pneumophila* |
|  | *Listeria monocytogenes* |
|  | *Mycobacterium avium* |
|  | *Mycobacterium intracellulare* |

**Note:** Some microbial markers have multiple ecological associations (e.g., *Aeromonas* spp. and *Vibrio* spp.); however, each marker was assigned to a single primary functional category for data visualization and statistical analyses to avoid duplication. Environmental source indicators were grouped separately because these organisms are primarily associated with environmental reservoirs, including water, soil, sediment, and biofilms, rather than with a specific human or animal source. Their occurrence in surface waters may reflect environmental persistence or transport through processes such as surface runoff, soil erosion, and sediment resuspension.
