# Supplementary material for: Urbanization shapes the landscape of antibiotic resistance genes and water quality microbial markers across an urban watershed
Source: Front Microbiol. 2026 Oct 9;17:1974267. doi: 10.3389/fmicb.2026.1974267 (PMC13690798; doi:10.3389/fmicb.2026.1974267)
Supplement: Supplementary file 4 [file Table_4.docx]

#### **Table S4.** Exploratory Pearson correlation coefficients between watershed characteristics, physicochemical parameters, ARG richness, and microbial marker richness.

| **Variable** | **ARG Richness** | **Microbial Marker Richness** |
| --- | --- | --- |
| Urbanization Rank | −0.410 | 0.073 |
| % Developed | **−0.480*** | 0.119 |
| % Forest | **0.528*** | −0.117 |
| % Cropland | 0.402 | −0.114 |
| Stream Order | −0.413 | 0.117 |
| Stream Density | **0.559*** | −0.097 |
| Elevation | **−0.469*** | 0.001 |
| Slope | **−0.548*** | 0.012 |
| Total Animals | −0.137 | 0.287 |
| Water Temperature | 0.309 | 0.282 |
| Water Depth | −0.036 | 0.296 |
| Precipitation | −0.015 | 0.226 |
| Dissolved Oxygen | −0.074 | −0.375 |
| pH | −0.401 | 0.451 |
| Conductivity | −0.123 | −0.191 |
| TDS | −0.123 | −0.126 |
| Turbidity | −0.193 | 0.042 |

**Note:** Pearson correlation coefficients were calculated using pooled site-year observations (n = 18). Watershed characteristics and total animal abundance were constant across sampling years for each site and are therefore interpreted as exploratory associations.
