# Supplementary material for: Urbanization shapes the landscape of antibiotic resistance genes and water quality microbial markers across an urban watershed
Source: Front Microbiol. 2026 Oct 9;17:1974267. doi: 10.3389/fmicb.2026.1974267 (PMC13690798; doi:10.3389/fmicb.2026.1974267)
Supplement: Supplementary file 5 [file Table_5.docx]

**Table S5.** Results of two-way ANOVA for physicochemical characteristics measured across the Rouge River watershed.

| **Variable** | **Source of variation** | **df** | **F** | ***P*-value** |
| --- | --- | --- | --- | --- |
| Water temperature (°C) | Site | 5 | 159.99 | <0.001 |
|  | Year | 2 | 1160.44 | <0.001 |
|  | Site x Year | 10 | 92.81 | <0.001 |
| Dissolved oxygen (mg L⁻¹) | Site | 5 | 18.77 | <0.001 |
|  | Year | 2 | 396.94 | <0.001 |
|  | Site x Year | 10 | 17.14 | <0.001 |
| pH | Site | 5 | 9.24 | <0.001 |
|  | Year | 2 | 60.46 | <0.001 |
|  | Site x Year | 10 | 12.07 | <0.001 |
| Electrical conductivity (µS cm⁻¹) | Site | 5 | 82.92 | <0.001 |
|  | Year | 2 | 536.41 | <0.001 |
|  | Site x Year | 10 | 53.48 | <0.001 |
| Total suspended solids (mg L⁻¹) | Site | 5 | 524.32 | <0.001 |
|  | Year | 2 | 2591.55 | <0.001 |
|  | Site x Year | 10 | 207.23 | <0.001 |
| Turbidity (NTU) | Site | 5 | 28.86 | <0.001 |
|  | Year | 2 | 6.41 | 0.004 |
|  | Site x Year | 10 | 14.94 | <0.001 |

**Note:** Two-way ANOVA was performed with sampling site and sampling year as fixed factors. F-statistics and associated *P*-values are shown for the main effects and their interaction. Significant Site × Year interactions indicate that spatial differences in physicochemical characteristics varied among sampling years.
